# Genomics of Dementia: *APOE*- and *CYP2D6*-Related Pharmacogenetics

**DOI:** 10.1155/2012/518901

**Published:** 2012-03-14

**Authors:** Ramón Cacabelos, Rocío Martínez, Lucía Fernández-Novoa, Juan C. Carril, Valter Lombardi, Iván Carrera, Lola Corzo, Iván Tellado, Jerzy Leszek, Adam McKay, Masatoshi Takeda

**Affiliations:** ^1^EuroEspes Biomedical Research Center, Institute for CNS Disorders and Genomic Medicine, EuroEspes Chair of Biotechnology and Genomics, Camilo José Cela University, 15165 Bergondo, Spain; ^2^Department of Psychiatry, Medical University of Wroclaw, Pasteura 10, 50-229 Wroclaw, Poland; ^3^Department of Psychiatry and Behavioral Sciences, Osaka University Graduate School of Medicine, 2-2 Yamadaoka, Suita-shi, Osaka 565-0871, Japan

## Abstract

Dementia is a major problem of health in developed societies. Alzheimer's disease (AD), vascular dementia, and mixed dementia account for over 90% of the most prevalent forms of dementia. Both genetic and environmental factors are determinant for the phenotypic expression of dementia. AD is a complex disorder in which many different gene clusters may be involved. Most genes screened to date belong to different proteomic and metabolomic pathways potentially affecting AD pathogenesis. The *ε*4 variant of the *APOE* gene seems to be a major risk factor for both degenerative and vascular dementia. Metabolic factors, cerebrovascular disorders, and epigenetic phenomena also contribute to neurodegeneration. Five categories of genes are mainly involved in pharmacogenomics: genes associated with disease pathogenesis, genes associated with the mechanism of action of a particular drug, genes associated with phase I and phase II metabolic reactions, genes associated with transporters, and pleiotropic genes and/or genes associated with concomitant pathologies. The *APOE* and *CYP2D6* genes have been extensively studied in AD. The therapeutic response to conventional drugs in patients with AD is genotype specific, with *CYP2D6*-PMs, *CYP2D6*-UMs, and *APOE-4/4* carriers acting as the worst responders. *APOE* and *CYP2D6* may cooperate, as pleiotropic genes, in the metabolism of drugs and hepatic function. The introduction of pharmacogenetic procedures into AD pharmacological treatment may help to optimize therapeutics.

## 1. Introduction

Senile dementia is a major health problem in developed countries and the primary cause of disability in the elderly. Alzheimer's disease (AD) is the most frequent form of dementia (50–70%), followed by vascular dementia (30–40%) and mixed dementia (15–20%). These prevalent forms of age-related neurodegeneration affect over 25 million people at present, and probably over 75 million people will be at risk in the next 20–25 years worldwide. The prevalence of dementia increases exponentially from approximately 1% at 60–65 years of age to over 30–35% in people older than 80 years. It is very likely that in those patients older than 75–80 years of age most cases of dementia are mixed in nature (degenerative + vascular), whereas pure AD cases are very rare after 80 years of age. The average annual cost per person with dementia ranges from €10,000 to €40,000, depending upon disease stage and country, with a lifetime cost per patient of over €150,000. In some countries, approximately 80% of the global costs of dementia (direct + indirect costs) are assumed by the patients and/or their families. About 10–20% of the costs in dementia are attributed to pharmacological treatment, including antidementia drugs, psychotropics (antidepressants, neuroleptics, and anxiolytics), and other drugs currently prescribed in the elderly (antiparkinsonians, anticonvulsants, vasoactive compounds, anti-inflammatory drugs, etc.). During the past 20 years over 300 drugs have been partially developed for AD, with the subsequent costs for the pharmaceutical industry, and only 5 drugs with moderate-to-poor efficacy and questionable cost-effectiveness have been approved in developed countries [1–3]. 

Dementia is a multifactorial/complex disorder where genetic, metabolic, vascular, and epigenetic factors interact along the lifespan leading to the premature death of neurons. With the advent of large-scale genomic studies, based on novel technology used for the mapping of the human genome, over 1,000 different genes have been screened over the past 20 years, but less than 100 genes have survived replication studies in different populations.

In recent times, significant advances have propelled the introduction of pharmacogenomic approaches in drug development and also in clinical practice to optimize therapeutics [4–8]. The vast majority of CNS drugs are metabolized via enzymes of the cytochrome P450 family (CYPs). The genes encoding *CYP2D6*, *CYP2C19*, *CYP2C9*, and *CYP3A4/5* isoenzymes are highly polymorphic, with great allelic variation in different ethnic groups. In the Western population, only 25% of its members are extensive metabolizers (EM) for the trigenic cluster integrated by CYPs *2D6 *+ *2C19 *+ *2C9*, the most relevant genes (and enzyme products) involved in drug metabolism, together with *CYP3A4/5*, which participates in the metabolism of over 80% of common drugs. The other 75% of the population is potentially at risk for developing adverse drug events (ADRs) due to defective variants encoding deficient enzymes which give rise to intermediate (IM), poor (PM), or ultrarapid metabolizers (UM). This population cluster of defective metabolizers requires dose adjustment to avoid side effects [5]. However, not only CYPs are important in terms of drug efficacy and safety. In fact, 5 categories of genes are mainly involved in pharmacogenomics: (i) genes associated with disease pathogenesis (e.g., *APP*,* PSEN1*,* PSEN2*,* MAPT*, and* APOE*) [4–6, 9, 10], (ii) genes associated with the mechanism of action of a particular drug (e.g., receptor genes) [11, 12], (iii) genes associated with phase I (CYPs) and phase II reactions (*UGTs*,* SULTs*,* GSTs*, and* NATs*) [10, 13–17], (iv) genes associated with transporters (*ABCs*, and* OATs*) [18–22], and (v) pleiotropic genes and/or genes associated with concomitant pathologies [23]. The *APOE* and *CYP2D6* genes have been extensively studied in AD. Both genes may influence pathogenesis and the pharmacogenetic outcome in patients with dementia.

## 2. Structural Genomics of Alzheimer's Disease

The genetic defects identified in AD can be classified into three main categories: (a) mendelian mutations in AD primary genes, (b) multiple susceptibility SNPs in many different genes distributed across the human genome, and (c) mitochondrial DNA (mtDNA) mutations.

 (a) Mendelian or mutational defects in genes are directly linked to AD, including (i) >30 mutations in the amyloid beta (A*β*) precursor protein (*APP*) gene (21q21) (AD1), (ii) >160 mutations in the presenilin 1 (*PSEN1*) gene (14q24.3) (AD3), and (iii) >10 mutations in the presenilin 2 (*PSEN2*) gene (1q31–q42) (AD4) [9, 24, 25]. *PSEN1 *and *PSEN2 *are important determinants of *β*-secretase activity responsible for proteolytic cleavage of APP and NOTCH receptor proteins. Mendelian mutations are very rare in AD (1 : 1000). Mutations in exons 16 and 17 of the *APP *gene appear with a frequency of 0.30% and 0.78%, respectively, in AD patients. Likewise, *PSEN1*, *PSEN2*, and microtubule-associated protein Tau (*MAPT*) (17q21.1) mutations are present in less than 2% of the cases. Mutations in these genes confer specific phenotypic profiles to patients with dementia: amyloidogenic pathology associated with *APP*, *PSEN1, *and *PSEN2 *mutations and tauopathy associated with MATP mutations, representing the two major pathogenic hypotheses for AD [9, 26–28].

 (b) Multiple polymorphic risk variants characterized in over 200 different genes can increase neuronal vulnerability to premature death [9]. Among susceptibility genes, the apolipoprotein E (*APOE*) gene (19q13.2) (AD2) is the most prevalent as a risk factor for AD, especially in those subjects harboring the *APOE-4 *allele, whereas carriers of the *APOE-2 *allele might be protected against dementia [9]. *APOE*-related pathogenic mechanisms are also associated with brain aging and with the neuropathological hallmarks of AD.

In 1993 Allen Roses and coworkers found a clear association between *APOE *genotypes and AD, demonstrating that the frequency of the *APOE-4 *allele was significantly higher in LOAD [29–31]. Since then, many other studies have confirmed the early findings of Saunders et al. [30, 31] and Corder et al. [32], reporting an increased frequency of the *APOE-4 *allele in AD and the association of the *APOE-4 *allele with LOAD and sporadic forms of AD [29–34]. *APOE-4 *may influence AD pathology interacting with APP metabolism and A*β* accumulation, enhancing the hyperphosphorylation of tau protein and NFT formation, reducing choline acetyltransferase activity, increasing oxidative processes, modifying inflammation-related neuroimmunotrophic activity and glial activation, altering lipid metabolism, lipid transport, and membrane biosynthesis in sprouting and synaptic remodeling, and inducing neuronal apoptosis [9, 29–37]. Age-related changes in brain structure and function have been reported as potential indicators of premature neurodegeneration [38].

Other genes of this category are included in Table 1. One of the newest members of the AD gene family is *SORL1*, a gene that encodes a mosaic protein with a domain structure which suggests it is a member of both the vacuolar protein sorting-10 (Vps10) domain-containing receptor family and the low-density lipoprotein receptor (LDLR). Inherited variants of the *SORL1* neuronal sorting receptor are associated with late-onset AD. Polymorphisms in two different clusters of intronic sequences within the *SORL1* gene may regulate tissue-specific expression of SORL1, which directs trafficking of APP into recycling pathways. When SORL1 is underexpressed, APP is sorted into A*β*-generating compartments leading to amyloid accumulation in neuronal tissues [39]. As with many other potential AD-related genes, the association of *SORL1* with AD [39, 40] could not be replicated in other studies [41].

Sorting protein-related receptor with A-type repeats (*SORLA*) is a major risk factor in cellular processes leading to AD. It acts as a sorting receptor for the APP that regulates intracellular trafficking and processing into amyloidogenic-beta peptides (A*β*). Overexpression of *SORLA* in neurons reduces while inactivation of gene expression accelerates amyloidogenic processing and senile plaque formation. Brain-derived neurotrophic factor (BDNF) is a major inducer of *SORLA* that activates receptor gene transcription through the ERK (extracellular regulated kinase) pathway. Expression of the receptor is significantly impaired in mouse models with genetic (*Bdn*(−/−)) or disease-related loss of BDNF activity in the brain (Huntington's disease). Exogenous application of BDNF reduced A*β* production in primary neurons and in the brain of wild-type mice *in vivo*, but not in animals genetically deficient for *Sorla*. According to these findings reported by Rohe et al. [43], the beneficial effects ascribed to BDNF in APP metabolism act through induction of *Sorla* which encodes a negative regulator of neuronal APP processing. The presence of the *BDNF *Val allele in itself and in combination with the *APOE-4 *allele can be risk factors for AD, Lewy body dementia, and Pick's disease [44].

Another interesting gene is *DHCR24* (3*β*-hydroxysterol-*δ*-24-reductase) or *Seladin-1*, a key element in the cholesterologenic pathway in which the DHCR24 enzyme catalyzes the transformation of desmosterol into cholesterol [45, 46]. *Seladin-1* was originally identified as a gene whose expression was downregulated in the AD brain, demonstrating a neuroprotective effect against neurodegeneration. Recent studies indicate that *Seladin-1*/*DHCR24* is an LXR (liver X nuclear hormone receptor) target gene potentially involved in the regulation of lipid raft formation [45].

Polymorphisms in the cholesteryl ester transfer protein (*CETP*) gene have been associated with exceptional longevity and lower cardiovascular risk, but associations with memory decline and dementia risk are unclear. Sanders et al. [47] tested the hypothesis that an SNP at *CETP* codon 405 (isoleucine to valine V405; SNP rs5882) is associated with a lower rate of memory decline and lower risk of incident dementia, including AD. Compared with isoleucine homozygotes, valine homozygotes had significantly slower memory decline and lower risk of dementia

Another gene, with potential therapeutic interest as a tau kinase, might be the *GSK3* gene. Analysis of the promoter and all 12 exons revealed that an intronic polymorphism (IVS2-68G>A) occurred at over twice the frequency among patients with frontotemporal dementia (10.8%) and patients with AD (14.6%) than in aged healthy subjects (4.1%). This is the first evidence that a gene known to be involved in tau phosphorylation is associated with risk for primary neurodegenerative dementias [48].

Promoter polymorphisms modulating HSPA5 expression might also increase susceptibility to AD. Endoplasmic reticulum chaperone heat shock 70 kDa protein 5 (HSPA5/GRP78) is known to be involved in APP metabolism and neuronal death in AD. Of the three major polymorphisms (−415G/A (rs391957), −370C/T (rs17840761), and −180Del/G (rs3216733)), the *HSPA5*-415G/A and −180Del/G variants showed significant differences between AD cases and controls. Subjects harboring the −415AA/−180GG genotype or the −415A/−180G allele might be less susceptible to developing AD [49].

The rs5952C and rs1568566T alleles of the *APOD* rs5952T/C and rs1568566C/T variants increase the risk for AD, whereas the rs5952T-rs1568566C haplotype reduces it [50]. ApoD is a lipoprotein-associated glycoprotein which is increased in the hippocampus and CSF of AD patients [50].

*CALHM1* encodes a multipass transmembrane glycoprotein that controls cytosolic Ca^2+^ concentrations and A*β* levels. The *CALHM1* P86L polymorphism (rs2986017) has been associated with AD [51].

Harold et al. [52] undertook a two-stage genome-wide association study (GWAS) of AD involving over 16,000 individuals and found association with SNPs at two loci not previously associated with the disease, at the *CLU* (Clusterine, *APOJ*) gene (rs11136000) and 5′ to the *PICALM* gene (rs3851179). In another GWAS with patients from France, Belgium, Finland, Italy, and Spain, Lambert et al. [53] found association with *CLU* and with the *CR1* gene, encoding the complement component (3b/4b) receptor 1 on chromosome 1 (rs6656401). Jun et al. [54] determined whether genotypes at *CLU*, *PICALM*, and *CR1* confer risk for AD and whether risk for AD associated with these genes is influenced by *APOE* genotypes in 7,070 cases with AD, 3,055 with autopsies, and 8,169 elderly cognitively normal controls, 1,092 with autopsies, from 12 different studies, including white, African American, Israeli-Arab, and Caribbean Hispanic individuals. They confirmed in a completely independent data set that *CR1* (rs3818361), *CLU* (rs11136000), and *PICALM* (rs3851179) are AD susceptibility loci in European ancestry populations. Genotypes at *PICALM* confer risk predominantly in *APOE-4*-positive subjects. Thus, *APOE* and *PICALM* synergistically interact. Two new loci were identified to have genome-wide significance for the first time: rs744373 near *BIN1* and rs597668 near *EXOC3L2/BLOC1S3/MARK4* [55].

Kramer et al. [56] conducted a GWAS to identify genetic mechanisms that distinguish nondemented elderly with a heavy NFT burden from those with a low NFT burden. Both a genotype test, using logistic regression, and an allele test provided consistent evidence that variants in the *RELN* gene are associated with neuropathology in the context of cognitive health. Immunohistochemical data for reelin expression in AD-related brain regions added support for these findings. Reelin signaling pathways modulate phosphorylation of tau, the major component of NFTs, either directly or through beta-amyloid pathways that influence tau phosphorylation. Upregulation of reelin may be a compensatory response to tau-related or beta-amyloid stress associated with AD even prior to the onset of dementia [56]. A*β* induces synaptic dysfunction in part by altering the endocytosis and trafficking of AMPA and NMDA receptors. Reelin is a neuromodulator that increases glutamatergic neurotransmission by signaling through the postsynaptic ApoE receptors ApoER2 and VLDLR and thereby potently enhances synaptic plasticity. Reelin can prevent the suppression of long-term potentiation and NMDA receptors, which is induced by levels of A*β* comparable to those present in an AD-afflicted brain. This reversal is dependent upon the activation of Src family tyrosine kinases. Durakoglugil et al. [57] proposed that A*β*, Reelin, and ApoE receptors modulate neurotransmission and thus synaptic stability as opposing regulators of synaptic gain control.

A variable-length, deoxythymidine homopolymer (poly-T) within intron 6 of the *TOMM40* gene was found to be associated with the age of onset of LOAD by Roses et al. [58]. This result was obtained with a phylogenetic study of the genetic polymorphisms that reside within the linkage disequilibrium (LD) block that contains the *TOMM40*, *APOE*, and *APOC1* genes from patients with LOAD and age-matched subjects without disease [59]. These new data explain the mean age at disease onset for patients with the *APOE4/4* genotype and differentiate two forms of *TOMM40* poly-T polymorphisms linked to APOE, with each form associated with a different age at disease onset distribution. When linked to APOE3 (encoding the *ε*3 isoform of APOE), the longer *TOMM40* poly-T repeats (19–39 nucleotides) at the rs10524523 (hereafter, 523) locus are associated with earlier age at onset and the shorter *TOMM40* 523 alleles (11–16 nucleotides) are associated with later age at onset. According to Roses [60], the data suggest that the poly-T alleles are codominant, with the age at onset phenotype determined by the two inherited 523 alleles, but with variable expressivity.

Ohe and Mayeda [61] reported that overexpression of high-mobility group A protein 1a (HMGA1a) causes aberrant exon 5 skipping of the presenilin-2 (*PSEN2*) pre-mRNA, which is almost exclusively detected in patients with sporadic AD. An electrophoretic mobility shift assay confirmed aberrant U1 small-nuclear-ribonucleoprotein-particle- (snRNP-) HMGA1a complex formation (via the U1-70 K component), with RNA containing a specific HMGA1a-binding site and an adjacent 5′ splice site. The HMGA1a-induced aberrant exon skipping is caused by impaired dissociation of U1 snRNP from the 5′ splice site, leading to a defect in exon definition.

Kelley et al. [62] characterized a kindred with a familial neurodegenerative disorder associated with a mutation in progranulin (*PGRN*). *PGRN* analysis revealed a single base pair deletion in exon 2 (c.154delA), which caused a frameshift (p.Thr52HisfsX2) and, therefore, creation of a premature termination codon and a likely null allele. In this large kindred, most affected individuals had clinical presentations that resembled AD or amnestic mild cognitive impairment associated with a mutation in *PGRN* and underlying frontotemporal lobar degeneration with ubiquitin-positive neuronal cytoplasmic and intranuclear inclusions (FTLD-U). Mutation in the *PGRN* gene can cause frontotemporal dementia (FTD9). Yu et al. [63] identified 58 genetic variants that included 26 previously unknown changes. 24 variants appeared to be pathogenic, including eight novel mutations. The frequency of *PGRN* mutations was 6.9% of all FTD-spectrum cases, 21.4% of cases with a pathological diagnosis of FTLD-U, 16.0% of FTD-spectrum cases with a family history of a similar neurodegenerative disease, and 56.2% of cases of FTLD-U with a family history. Haploinsufficiency of *PGRN* is the predominant mechanism leading to FTD.

Polymorphisms within the promoter region of the vascular endothelial growth factor (*VEGF*) gene might elevate the risk for AD. In a Tunisian population, Smach et al. [64] found that the distribution of genotype and allele frequencies of the *VEGF* (−2578C/A) and (−1154G/A) polymorphisms did not differ significantly between AD and control groups. In the subgroup of *APOE-4* carriers, the −2578A was observed to be significantly higher in the AD patients than in the control individuals.

Endothelin-converting enzyme (*ECE-1*) is also a candidate AD susceptibility gene. Individuals homozygous for the C-338A polymorphism (AA) within the *ECE1* gene promoter region are at reduced risk of developing late-onset AD (LOAD). A further polymorphism, T-839G, is present within the *ECE1* promoter region but there is no significant association between T-839G and LOAD; however, the combined 839T/338A haplotype is associated with decreased risk of LOAD, suggesting that the *ECE1* 338A allele is protective against LOAD in a Chinese population [65].

Downregulation of somatostatin (SST) expression in the human brain during early stages of aging may lead to an elevation in the steady-state levels of A*β* and therefore may be involved in AD progression. Alterations in the *SST* gene might alter its expression or function and also play a role in the pathogenesis of sporadic AD (SAD). C/T polymorphisms (rs4988514) were screened in the 3′ untranslated region of the *SST* gene. The C allele of the rs4988514 polymorphism had an increased incidence in the SAD group compared to the control group in the Chinese population. In subjects with the *APOE-4* allele, the presence of both the CC genotype and the C allele of this polymorphism was elevated in the SAD group compared to the control group. The C allele of the rs4988514 polymorphism may increase the risk for AD in the Chinese population and possibly have additive effect with the *APOE-4* allele [66].

The receptor for advanced glycation end products (RAGE) is associated with several pathological states including AD pathology, while its soluble form (sRAGE) acts as a decoy receptor. Li et al. [67] studied an SNP in the *RAGE* gene (G82S; rs2070600) and an SNP associated with increased ligand affinity of *RAGE*. Analysis of a Chinese cohort showed a higher prevalence of the *RAGE* 82S allele and GS + SS genotype in EOAD patients. RAGE contributes to transport of A*β* from the cell surface to the intracellular space. Pretreatment of cultured neurons from wild-type mice with neutralizing antibody to RAGE and neurons from *Rage* knockout mice displayed decreased uptake of A*β* and protection from A*β*-mediated mitochondrial dysfunction [68].

The TAR-DNA-binding protein (TDP-43) has been postulated as the disease protein in amyotrophic lateral sclerosis and frontotemporal lobar dementia with ubiquitin-positive inclusions. TDP-43 may also play a role in the pathogenesis of AD. Shibata et al. [69] identified an association between a specific haplotype (G-A-A-G) of the *TDP-43* gene and risk for AD.

Immune dysfunction and aberrant inflammatory reactions are present in AD neuropathology. Neurons express enzymes such as cyclooxygenases (COXs) and 5-lipoxygenase (5-LO), which are considered important in inflammatory cells. It has been suggested that COX-2 and 5-LO enzymes may play a role in the pathophysiology of AD. A significant difference was observed in the distribution of the −765G *COX-2* and −1708A *5-LO* alleles between AD cases and controls. *COX-2* −765G and *5-LO* −1708A alleles were overrepresented in AD patients and underrepresented in controls [70]. The HLA-A*01 variant might also be associated with AD [71]. SNPs in the regulatory regions of the cytokine genes for tumor necrosis factor alpha (*TNF-alpha*), interleukin- *(IL-) 6,* and *IL-10* have been suggested to influence the risk of AD with conflicting results. Heterozygotes (AG) or combined genotype (AG + AA) for *IL-10* −1082 was associated with an approximately two-fold increase in the risk of AD. Carriers of A alleles of both *TNF-alpha*-308 and *IL-10* −1082 had 6.5 times higher risk for AD in comparison with noncarriers. Concomitant presence of both mutant *TNF*-*alpha*-308 A and *IL-6* −174 C alleles raised three-fold the AD risk, whereas there was no notable risk for AD afflicted by *IL-6 −*174 polymorphism alone [72, 73]. Interleukin-33 (*IL-33*), a newly described member of the *IL-1* family, is located on chromosome 9p24, a chromosomal region of interest in AD. Three intronic rs1157505, rs11792633, and rs7044343 SNPs within *IL-33* have been reported to be associated with risk of AD in Caucasian and Chinese populations [74].

Aromatase gene polymorphisms have also been associated with AD [75]. Polymorphisms in genes encoding amyloid-beta-peptide- (A*β*-) degrading enzymes neprilysin (NEP) and insulin-degrading enzyme (IDE) individually affect the susceptibility to AD among the Finnish population [76]. Nicastrin (NCSTN) is a type I transmembrane glycoprotein and an essential component of *γ*-secretase, a multiprotein complex required for the production of the mature form of A*β*. Overexpression of wild-type *NCSTN* increases A*β* production, indicating that the strict regulation of *NCSTN* expression may play a fundamental role in the pathogenesis of AD. Zhong et al. [77] investigated the effect of an SNP (rs10752637) located in the promoter region of the *NCSTN* gene, on *NCSTN* promoter activity. The distributions of the rs10752637 genotypes and allele frequencies were significantly different between the AD and control groups, with the −922T allele significantly associated with the occurrence of AD. Reporter assays indicated that the rs10752637 −922T allele had a significantly increased promoter activity relative to the −922G allele. The rs10752637 SNP can probably influence the expression of *NCSTN*, and this may be an influencing factor during the pathogenesis of AD.

The FISH (five SH3 domains) adapter protein and ADAM12 (a disintegrin and metalloprotease) may mediate the neurotoxic effect of A*β*. Both genes are located on chromosome 10, within a region linked to AD (for *SH3PXD2A*) or nearby (for *ADAM12*). Two variants of these genes (rs3740473 for *SH3PXD2A* and rs11244787 for *ADAM12*) have been associated with increased risk for developing AD, but these findings could not be confirmed in different populations [78].

Paraoxonase 1 (*PON1*) L55M and Q192R genetic variants might affect individual susceptibility to environmental events, such as exposure to cholinesterase inhibitors. The L55M Met allele exerts an AD risk-enhancing effect only in men, whereas both men and women carrying the M55M/Q192Q genotype exhibit increased survival and later age of onset. These genetic variants are also individually and significantly associated, sometimes in opposite directions for both genders, with beta-amyloid levels, senile plaque accumulation, and choline acetyltransferase activity in brain areas [79].

Liu et al. [80] studied the potential association of polymorphisms in NMDA receptor subunits NR3A and NR3B, encoded by the *GRIN3A* and *GRIN3B* genes, with AD, on the basis that memantine, an N-methyl-D-aspartate (NMDA) receptor antagonist, may provide some clinical benefit in AD patients. Two SNPs, 3104G/A (rs10989563) and 3723G/A (rs3739722), in the *GRIN3A* gene, and two *GRIN3B* gene polymorphisms, 1210C/T (rs4807399) and 1730C/T (rs2240158), were analyzed. Upon genotyping of the exonic polymorphism in the *GRIN3A* gene, the G allele was present at a higher rate than the A allele at position 3723 in AD patients compared with normal groups. Three haplotypes (designated Ht1-3) were identified from these two polymorphisms (3104G/A and 3723G/A), and the distribution of Ht2 (AG) differed between AD patients and controls. The two *GRIN3B* gene variants 1210C/T and 1730C/T did not show association with AD. These observations suggest that the genetic variation of the NR3A, but not NR3B, subunit of the NMDA receptor may be a risk factor for AD pathogenesis among the Taiwanese population. Di Maria et al. [81] reported that the occurrence of delusions and hallucinations in AD is associated with variations in the *G72/DAOA* gene (rs2153674), which is supposed to play a key role in the glutamate pathway regulated through the NMDA receptors. Martínez et al. [82] studied the influence of the catechol-*O*-methyltransferase (*COMT*) gene (polymorphism Val158 Met) as a risk factor for AD and mild cognitive impairment of the amnesic type (MCI) and its synergistic effect with *APOE* variants in the Basque Country. Neither *COMT* alleles nor genotypes were independent risk factors for AD or MCI; however, the high activity genotypes (GG and AG) showed a synergistic effect with the *APOE-4* allele, increasing the risk of AD.

Peptidyl-prolyl isomerase NIMA-interacting 1 (*PIN1*) plays a significant role in the brain and is implicated in numerous cellular processes related to AD and other neurodegenerative conditions. Analysis of 18 *PIN1* common polymorphisms and their haplotypes in EOAD, LOAD, and FTD individuals in comparison with the control group did not reveal their contribution to disease risk. In six unrelated familial AD patients four novel *PIN1* sequence variants were detected. The c.58+64C>T substitution identified in three patients was located in an alternative exon. *In silico* analysis suggested that this variant highly increases a potential affinity for a splicing factor and introduces two intronic splicing enhancers. In the peripheral leukocytes of one living patient carrying the variant, a 2.82-fold decrease in PIN1 expression was observed [83].

Alzheimer's and prion diseases are neurodegenerative disorders characterized by the abnormal processing of A*β* peptide and prion protein (PrP^C^), respectively. PrP^C^ may play a critical role in the pathogenesis of AD. PrP^C^ interacts with and inhibits the *β*-secretase BACE1, the rate-limiting enzyme in the production of A*β*. PrP^C^ was also identified as a receptor for A*β* oligomers, and the expression of PrP^C^ appears to be controlled by the amyloid intracellular domain (AICD). PrP^C^ exerts an inhibitory effect on BACE1 to decrease both A*β* and AICD production, and the AICD upregulates PrP^C^ expression, thus maintaining the inhibitory effect of PrP^C^ on BACE1. According to Kellett and Hooper [84], this feedback loop is disrupted in AD, and the increased level of A*β* oligomers binds to PrP^C^ and prevents it from regulating BACE1 activity. It is also likely that *PRNP* gene mutations contribute to AD pathogenesis [9]. *HECTD2* maps to 10q and has been implicated in susceptibility to human prion disease. A *HECTD2* intronic tagging SNP, rs12249854 (A/T), has been studied in AD. The rs12249854 minor allele (A) frequency was higher (5.8%) in AD as compared to controls (3.9%). No significant difference was seen in minor allele frequency in the presence or absence of the *APOE-4* allele. According to these results, it appears that the common haplotypes of *HECTD2*, tagged by rs12249854, are not associated with susceptibility to LOAD [85].

Ubiquitin-conjugating enzyme E2I (Ubc9) ligates small ubiquitin-related modifier (SUMO) to target proteins, resulting in changes of their localization, activity, or stability. Sumoylation of APP was reported to be associated with decreased levels of A*β* aggregates, suggesting that sumoylation may play a role in the pathogenesis of AD. Ahn et al. [86] investigated the association between genetic variations of Ubc9 gene (*UBE2I*) and LOAD in Koreans. The genotype distribution of a polymorphism in intron 7 (rs761059) differed between AD cases and controls and one haplotype (ht2 CAGAG) was found in 14.0% of the AD patients and in 11.1% of the controls. Stratification by the *APOE-4 *allele gave no significant difference between the groups. When the samples were stratified by gender, the genotypes of two SNPs (rs8052688, rs8063) were significantly associated with the risk of MCI among women.

To gain insights into the evolution of the regulatory mechanisms of the aged brain, Persengiev et al. [87] compared age-related differences in microRNA (miRNA) expression levels in the cortex and cerebellum of humans, chimpanzees, and rhesus macaques on a genome-wide scale. In contrast to global miRNA downregulation, a small subset of miRNAs was found to be selectively upregulated in the aging brain of all three species. miR-144 appeared to be associated with the aging progression. miR-144 plays a central role in regulating the expression of ataxin 1 (*ATXN1*), a gene which is associated with spinocerebellar ataxia type 1 (SCA1). miRNA activity, including miR-144, -101, and -130 processing, was increased in the cerebellum and cortex of SCA1 and Alzheimer's patients relative to healthy aged brains. The activation of miRNA expression in the aging brain might serve to reduce the cytotoxic effect of polyglutamine expanded *ATXN1* and the deregulation of miRNA expression might be a risk factor for neurodegeneration. Bettens et al. [88] also obtained evidence for association between rs179943, an intronic SNP in *ATXN1* at 6p22.3, and AD.

The cholesterol transporter ATP-binding cassette transporter A1 (*ABCA1*) moves lipids onto apolipoproteins including ApoE. Donkin et al. [89] reported that in amyloid mouse models of AD, ABCA1 deficiency exacerbates amyloidogenesis, whereas ABCA1 overexpression ameliorates amyloid load, suggesting a role for *ABCA1* in A*β* metabolism. Agonists of liver X receptors (LXRs), including GW3965, induce transcription of several genes including *ABCA1* and *ApoE*, reduce A*β* levels, and improve cognition in AD mice. Treatment of *APP*/*PS1* mice with GW3965 increased ABCA1 and ApoE protein levels. *ABCA1* was observed to require significantly elevated ApoE levels in brain tissue and CSF upon GW3965 treatment. *APP*/*PS1* mice treated with either 2.5 mg/kg/d or 33 mg/kg/d of GW3965 showed a clear trend toward reduced amyloid burden in hippocampus and whole brain, whereas treated *APP*/*PS1* mice lacking *ABCA1* failed to display reduced amyloid load in whole brain and showed trends toward increased hippocampal amyloid. Treatment of *APP*/*PS1* mice with either dose of GW3965 completely restored novel object recognition (NOR) memory to wild-type levels, which required ABCA1. These results reported by Donkin and coworkers suggest that *ABCA1* contributes to several beneficial effects of the LXR agonist GW3965 in *APP*/*PS1* mice.

The phospholipid transfer protein (PLTP) reduces phosphorylation of tau in human neuronal cells. Patients with AD have significantly higher levels of PLTP in brain tissue and significantly lower PLTP-mediated phospholipid transfer activity in cerebrospinal fluid. PLTP also affects ApoE secretion from glial cells. Kuerban et al. [90] investigated whether SNPs of the *PLTP* gene are associated with AD in the Japanese population and found no genetic association between *PLTP* and AD.

Genome-wide association studies (GWASs) in AD highlight over two dozen novel potential susceptibility loci beyond the well-established *APOE* association, including *GAB2* (GRB2-associated binding protein 2), galanin-like peptide (*GALP*), piggyBac transposable element derived 1 (*PGBD1*), tyrosine kinase, non-receptor 1 (*TNK1*), and at least three replicated loci in hitherto uncharacterized genomic intervals on chromosomes 14q32.13, 14q31.2, and 6q24.1, probably implicating the existence of novel AD genes in these regions [91].

(c) Diverse mutations located in mitochondrial DNA (mtDNA) through heteroplasmic transmission can influence aging and oxidative stress conditions, conferring phenotypic heterogeneity [9, 92]. The human presequence protease (hPreP) was recently shown to be the major mitochondrial A*β*-degrading enzyme. Genetic variation in the hPreP gene *PITRM1* has been investigated by Pinho et al. [93]. No genetic association was found between any of the SNPs and the risk for AD; however, functional analysis of four nonsynonymous SNPs in hPreP revealed a decreased activity compared to wild-type hPreP. Using A*β*, the presequence of ATP synthase F1*β* subunit and a fluorescent peptide as substrates, the lowest activity was observed for the hPreP(A525D) variant, corresponding to rs1224893, which displayed only 20–30% of wild-type activity. Genetic variation in the hPreP gene *PITRM1* might contribute to mitochondrial dysfunction in AD.

Recent data suggest the possible contribution of heme deficiency to the progressive derangement of mitochondria in the AD brain; shortage of heme, and particularly of heme-a, actually leads to loss of mitochondrial cytochrome c oxidase (COX), abnormal production of reactive oxygen species, and altered amyloid precursor protein metabolism. Differences in the amount and/or functioning of COX assembly subunits 10 (COX10) and 15 (COX15), the key enzymes involved in heme-a biosynthesis, could be linked to variations of the individual risk to develop AD. Vitali et al. [94] analyzed mRNA expression in the hippocampus from AD patients and controls, as well as nucleotide variations in DNA sequences in AD. *COX15* mRNA was significantly more abundant in the cerebral tissue of AD patients, and the IVS-178G>AN SNP in *COX10* and the c+1120C>T SNP in *COX15* were significantly less represented in AD, suggesting a possible protective role.

Japanese AD patients are associated with the haplogroups G2a, B4c1, and N9b1. Takasaki [95] compared mitochondrial haplogroups of AD patients with those of other classes of Japanese (centenarians, Parkinson's disease (PD), type 2 diabetes mellitus (T2D), and nonobese young males). The four classes of people were associated with the following haplogroups: Japanese centenarians with M7b2, D4b2a, and B5b; PD patients with M7b2, B4e, and B5b; T2D patients with B5b, M8a1, G, D4, and F1; Japanese healthy nonobese young males with D4g and D4b1b. The haplogroups of the AD patients differed from those of the other four categories.

Santoro et al. [96] applied for the first time a high-resolution analysis to investigate the possible association between mtDNA-inherited sequence variation and AD in 936 AD patients and 776 cognitively assessed normal controls from central and northern Italy. Among over 40 mtDNA subhaplogroups analyzed, they found that sub-haplogroup H5 is a risk factor for AD, particularly in females, independently of the *APOE* genotype. The H5a subgroup of molecules, harboring the 4336 transition in the *tRNAGln* gene, was about threefold more represented in AD patients than in controls (2.0% versus 0.8%), and it might account for the increased frequency of H5 in AD patients (4.2% versus 2.3%). The complete resequencing of the 56 mtDNAs belonging to H5 revealed that AD patients showed a trend towards a higher number of sporadic mutations in tRNA and rRNA genes when compared with controls.

**Gene Interactions**

Although *APP* and *PSEN* mutations are considered causative factors for AD, the total number of mutations identified in the *APP*, *PSEN1,* and *PSEN2* genes account for less than 3% of the cases with AD, clearly indicating that neurodegeneration associated with AD pathogenesis cannot be exclusively attributed to *APP/PSEN*-related cascades (amyloid hypothesis). Alterations in the ubiquitin-proteasome system and biochemical disarray in the chaperone machinery are alternative and/or complementary pathogenic events potentially leading to defects in protein synthesis, folding, and degradation with subsequent conformational changes, aggregation, and accumulation in cytotoxic deposits [4, 9]. A more plausible explanation would seem to be that multiple susceptibility SNPs with a very subtle genetic variation cooperatively contribute, in concert with environmental factors and concomitant CNS vulnerability, to premature neurodegeneration in dementia.

We have compared the distribution and frequency of major polymorphic variants of different genes potentially associated with AD (i.e., *APOE*, *PSEN1*, *A2M*-V1001, *A2M*-I/D, *ACE*, *FOS*, *AGT*-235, *AGT*-174, *eNOS3*-E298D, *eNOS3-*27bpTR, *CETP*, and *MTHFR*) in the general population, in adults (>45 years) with no family history of dementia, and in patients with dementia, and could not find any significant differences among the three groups except in the case of the *APOE* gene, which exhibits a clear accumulation of *APOE-3/4* and *APOE-4/4* genotypes (overload of the *APOE-4* allele) in AD cases [5]. If we consider that a genetic variation higher than 2% could be of significant value, then several polymorphisms clearly differ in AD as compared with the other two population clusters, including the *PSEN1*-1/2, *ACE*-D/D, *ACE*-I/I, *CEPT*-B1/B1, and *MTHFR*-T/T polymorphisms [5].

Defective functions of genes associated with longevity may influence premature neuronal survival, since neurons are potential pacemakers defining lifespan in mammals [9, 97]. Hypothalamic expression of CREB-binding protein (*CBP*) and CBP-binding partner special AT-rich sequence binding protein 1 (*SATB-1*) is highly correlated with lifespan across five strains of mice, and expression of these genes decreases with age and diabetes in mice. In a transgenic A*β*42 model of AD, cbp-1 RNAi prevents protective effects of bacterial dilution (bDR) and accelerates A*β*42-related pathology. Consistent with the function of CBP as a histone acetyltransferase, drugs that enhance histone acetylation increase lifespan and reduce A*β*42-related pathology, protective effects completely blocked by cbp-1 RNAi. Other factors implicated in lifespan extension are also *CBP*-binding partners, suggesting that *CBP* constitutes a common factor in the modulation of lifespan and disease burden by DR and the insulin/*IGF1* signaling pathway [98].

AD patients have been reported to have shorter telomeres in peripheral blood leukocytes (PBLs) than age-matched control subjects. However, it is unclear if PBL telomere length reflects brain telomere length, which might play a more direct role in AD pathogenesis. Lukens et al. [99] examined the correlation between PBL and cerebellum telomere length in AD patients. The PBL and cerebellum telomere lengths were directly correlated in individuals with AD. Nonetheless, cerebellum telomere lengths were not significantly different in AD patients and age-matched control subjects. Reduced PBL telomere length in AD might not reflect reduced telomere length in bulk brain tissue but may be a marker of changes in a subset of brain tissues or other tissues that affect the pathogenesis of AD. Zekry et al. [100] evaluated the usefulness of telomere length alone or combined with *APOE* polymorphism in diagnosing mild cognitive impairment (MCI) and in differentiating AD from vascular and mixed dementia. Although *APOE-4* was associated with dementia, no significant differences in telomere length were found among patients with different types of dementia. The combination of telomere length and *APOE-4* did not confer a significantly higher dementia risk [100].

## 3. Functional Genomics

Over 80% of the genes that conform the structural architecture of the human genome are expressed in the brain in a time-dependent manner along the lifespan. The cellular complexity of the CNS (with 10^3^ different cell types) and synapses (with each of the 10^11^ neurons in the brain having around 10^3^-10^4^ synapses with a complex multiprotein structure integrated by 10^3^ different proteins) requires a very powerful technology for gene expression profiling, which is still in its very early stages and is not devoid of technical obstacles and limitations [101]. Transcripts of 16,896 genes have been measured in different CNS regions. Each region possesses its own unique transcriptome fingerprint which is independent of age, gender, and energy intake. Less than 10% of genes are affected by age, diet, or gender, with most of these changes occurring between middle and old age. Gender and energy restriction have robust influences on the hippocampal transcriptome of middle-aged animals. Prominent functional groups of age- and energy-sensitive genes are those encoding proteins involved in DNA damage responses, mitochondrial and proteasome functions, cell fate determination, and synaptic vesicle trafficking. The systematic transcriptome dataset provides a window into mechanisms of neuropathogenesis and CNS vulnerability [102].

Functional genomics studies have demonstrated the influence of many genes on AD pathogenesis and phenotype expression. The study of genotype-phenotype correlations is essential for the evaluation of the actual impact of specific polymorphic variants of a particular gene on the clinical manifestation of the disease and/or biological markers reflecting the disease condition or different biological states of the individual. Mutations in the *APP*, *PSEN1*, *PSEN2*, and *MAPT* genes give rise to well-characterized differential neuropathological and clinical phenotypes of dementia [9, 24, 25]. *APP* mutations are associated with AD1, early-onset progressive autosomal recessive dementia, early-onset AD with cerebral amyloid angiopathy, and hereditary amyloidosis with cerebral hemorrhage Dutch type, Italian type, or Iowa type. *PSEN1* mutations are associated with the phenotypes of familial AD3, familial AD3 with unusual plaques, familial AD with spastic paraparesis and unusual plaques, familial AD with paraparesis and apraxia, frontotemporal dementia, Pick's disease, and dilated cardiomyopathy. *MAPT* mutations are associated with frontotemporal dementia, frontotemporal dementia with parkinsonism, Pick's disease, progressive supranuclear palsy, progressive atypical supranuclear palsy, tauopathy, and respiratory failure [9].

Transgenic animals also reproduce to some extent the neuropathological hallmarks of AD in a sequential manner. The triple transgenic mouse model of AD (3xTg-AD) harbors three AD-related loci: human PS1M146V, human APPswe, and human MAPTP301L. These animals develop both amyloid plaques and NFT-like pathology in a progressive and age-dependent manner in hippocampus, amygdala, and cerebral cortex, the main foci of human AD neuropathology. The evolution of AD-related transgene expression, amyloid deposition, tau phosphorylation, astrogliosis, and microglia activation throughout the hippocampus, entorhinal cortex, primary motor cortex, and amygdala over a 26-month period has been immunohistochemically documented. Intracellular A*β* accumulation is the earliest of AD-related pathologies to be detectable, followed temporally by phospho-tau, extracellular A*β*, and finally paired helical filament and NFT pathology [103]. In the same model, a decrease in neurogenesis directly associated with the presence of amyloid plaques and an increase in the number of A*β* containing neurons in the hippocampus has been demonstrated [104].

Different *APOE* genotypes also confer specific phenotypic profiles to AD patients. Some of these profiles may add risk or benefit when the patients are treated with conventional drugs, and in many instances the clinical phenotype demands the administration of additional drugs which increase the complexity of therapeutic protocols. From studies designed to define *APOE*-related AD phenotypes [4–9, 37, 97, 105–114], several confirmed conclusions can be drawn: (i) the age at onset is 5–10 years earlier in approximately 80% of AD cases harboring the *APOE-4/4* genotype; (ii) the serum levels of ApoE are lowest in *APOE-4/4*, intermediate in *APOE-3/3* and *APOE-3/4*, and highest in *APOE-2/3* and *APOE-2/4*; (iii) serum cholesterol levels are higher in *APOE-4/4* than in the other genotypes; (iv) HDL-cholesterol levels tend to be lower in *APOE-3 *homozygotes than in *APOE-4* allele carriers; (v) LDL-cholesterol levels are systematically higher in *APOE-4/4* than in any other genotype; (vi) triglyceride levels are significantly lower in *APOE-4/4*; (vii) nitric oxide levels are slightly lower in *APOE-4/4*; (viii) serum A*β* levels do not differ between *APOE-4/4 *and the other most frequent genotypes (*APOE-3/3*, *APOE-3/4*); (ix) blood histamine levels are dramatically reduced in *APOE-4/4* as compared with the other genotypes; (x) brain atrophy is markedly increased in *APOE-4/4 *> *APOE-3/4 *> *APOE-3/3*; (xi) brain mapping activity shows a significant increase in slow wave activity in *APOE-4/4* from early stages of the disease; (xii) brain hemodynamics, as reflected by reduced brain blood flow velocity and increased pulsatility and resistance indices, is significantly worse in *APOE-4/4 *(and in *APOE-4* carriers, in general, as compared with *APOE-3* carriers); (xiii) lymphocyte apoptosis is markedly enhanced in *APOE-4 *carriers; (xiv) cognitive deterioration is faster in *APOE-4/4* patients than in carriers of any other *APOE *genotype; (xv) occasionally, in approximately 3–8% of the AD cases, the presence of some dementia-related metabolic dysfunctions (e.g., iron, folic acid, and vitamin B_12_ deficiencies) accumulates more in *APOE-4 *carriers than in *APOE-3 *carriers; (xvi) some behavioral disturbances (bizarre behaviors and psychotic symptoms), alterations in circadian rhythm patterns (e.g., sleep disorders), and mood disorders (anxiety and depression) are slightly more frequent in *APOE-4* carriers; (xvii) aortic and systemic atherosclerosis is also more frequent in *APOE-4* carriers; (xviii) liver metabolism and transaminase activity also differ in *APOE-4/4* with respect to other genotypes; (xix) blood pressure (hypertension) and other cardiovascular risk factors also accumulate in *APOE-4*; (xx) *APOE-4/4* are the poorest responders to conventional drugs. These 20 major phenotypic features clearly illustrate the biological disadvantage of *APOE-4* homozygotes and the potential consequences that these patients may experience when they receive pharmacological treatment [4–7, 9, 28, 37, 97, 105–118].

## 4. Dementia Phenotype and Biomarkers

The phenotypic features of the disease represent the biomarkers to be modified with an effective therapeutic intervention. Important differences have been found in the AD population as compared with healthy subjects in different biological parameters, including blood pressure, glucose, cholesterol, and triglyceride levels, transaminase activity, hematological parameters, metabolic factors, thyroid function, brain hemodynamic parameters, and brain mapping activity [4, 5, 9, 97, 105, 108–111]. Blood pressure values, glucose levels, and cholesterol levels are higher in AD than in healthy elderly subjects. Approximately 20% of AD patients are hypertensive, 25% are diabetics, 50% are hypercholesterolemic, and 23% are hypertriglyceridemic. Over 25% of the patients exhibit high GGT activity, 5–10% show anemic conditions, 30–50% show an abnormal cerebrovascular function characterized by poor brain perfusion, and over 60% have an abnormal electroencephalographic pattern, especially in frontal, temporal, and parietal regions, as revealed by quantitative EEG (qEEG) or computerized mapping [5, 9, 105]. Significant differences are currently seen between females and males, indicating the effect of gender on the phenotypic expression of the disease. In fact, the prevalence of dementia is 10–15% higher in females than in males from 65 to 85 years of age. All these parameters are highly relevant when treating AD patients because some of them reflect a concomitant pathology that also needs therapeutic consideration. They can also represent general biomarkers together with regional brain atrophy and perfusion and cognitive function, which may serve as therapeutic outcome measures. Other biomarkers of potential interest include cerebrospinal fluid (CSF) and peripheral levels of A*β*42, protein tau, histamine, interleukins, and some other candidate markers [5, 119, 120]. In proteomic studies, several candidate CSF protein biomarkers have been assessed in neuropathologically confirmed AD, nondemented (ND) elderly controls, and non-AD dementias (NADDs). Markers selected included apolipoprotein A-1 (ApoA1), hemopexin (HPX), transthyretin (TTR), pigment epithelium-derived factor (PEDF), A*β*1–40, A*β*1–42, total tau, phosphorylated tau, *α*-1 acid glycoprotein (A1GP), haptoglobin, zinc *α*-2 glycoprotein (Z2GP), and apolipoprotein E (ApoE). The concentrations of A*β*1–42, ApoA1, A1GP, ApoE, HPX, and Z2GP differed significantly among AD, ND, and NADD subjects. The CSF concentrations of these three markers distinguished AD from ND subjects with 84% sensitivity and 72% specificity, with 78% of subjects correctly classified. By comparison, using A*β*1–42 alone gave 79% sensitivity and 61% specificity, with 68% of subjects correctly classified. For the diagnostic discrimination of AD from NADD, only the concentration of A*β*1-42 was significantly related to diagnosis, with a sensitivity of 58% and a specificity of 86% [121].

## 5. Therapeutic Strategies in Dementia

Modern therapeutic strategies in AD are addressed to interfering with the main pathogenic mechanisms potentially involved in AD. Major pathogenic events (drug targets) and their respective therapeutic alternatives include the following: genetic defects, *β*-amyloid deposition, tau-related pathology, apoptosis, neurotransmitter deficits, neurotrophic deficits, neuronal loss, neuroinflammation, oxidative stress, calcium dysmetabolism, neuronal hypometabolism, lipid metabolism dysfunction, cerebrovascular dysfunction, neuronal dysfunction associated with nutritional and/or metabolic deficits, and a miscellany of pathogenic mechanisms potentially manageable with diverse classes of chemicals or biopharmaceuticals [4, 5, 9, 37, 97, 105–112, 115]. Since the early 1980s, the neuropharmacology of AD was dominated by the acetylcholinesterase inhibitors, represented by tacrine, donepezil, rivastigmine, and galantamine [2, 3, 122]. Memantine, a partial NMDA antagonist, was introduced in the 2000s for the treatment of severe dementia [123], and the first clinical trials with immunotherapy, to reduce amyloid burden in senile plaques, were withdrawn due to severe ADRs [124, 125]. During the past few years no relevant drug candidates have been postulated for the treatment of AD, despite the initial promises of *β*- and *γ*-secretase inhibitors [4, 126, 127]. However, assuming that the best treatment for AD is neuronal death prevention prior to the onset of the disease, novel therapeutic options and future candidate drugs for AD might be a new generation of anti-amyloid vaccines, such as DNA A*β*42 trimer immunization [128], heterocyclic indazole derivatives (inhibitors of the serum- and glucocorticoid-inducible-kinase 1 (*SGK1*)) [129], NSAID-like compounds [130], IgG-single chain Fv fusion proteins [131], Hsp90 inhibitors and HSP inducers [132], inhibitors of class I histone deacetylases [133], some phenolic compounds [134], agonists of the peroxisome proliferator-activated receptor-gamma (*PPAR-gamma*) [135], microRNAs [136], and gene silencing (RNAi) [137].

## 6. Pharmacogenetics of AD-Related Genes

**Figure 1:**
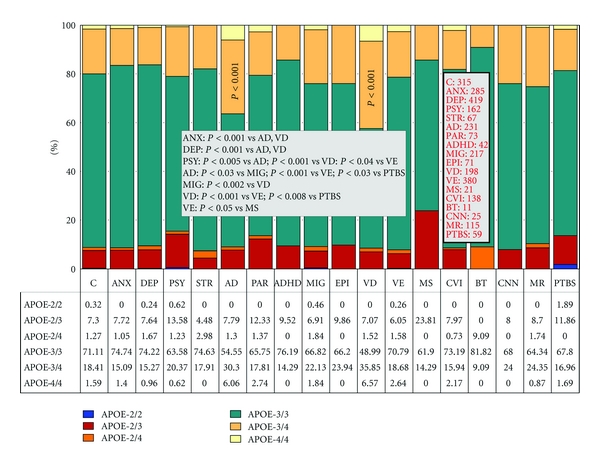
Distribution and frequency of *APOE* genotypes in patients with CNS disorders. C: controls; ANX: anxiety; DEP: depression; PSY: psychotic disorders; STR: stroke; AD: Alzheimer's disease; PAR: Parkinson's disease; ADHD: attention-deficit hyperactivity disorder; MIG: migraine; EPI: epilepsy; VD: vascular dementia; VE: vascular encephalopathy; MS: multiple sclerosis; CVI: cerebrovascular insufficiency; BT: brain tumors; CNN: cranial nerve neuropathies; MR: mental retardation; PTBS: posttraumatic brain injury syndrome. Source: R. Cacabelos. CIBE Database. EuroEspes Biomedical Research Center, Institute for CNS Disorders, Coruña, Spain.

Over 500 studies reported during the past two decades have postulated the potential involvement of *APOE* in dementia and other CNS disorders [9]. The distribution and frequency of *APOE* genotypes (Figure 1) have been investigated in 315 Spanish controls with no family history of neuropsychiatric disorders and in patients with anxiety, depression, psychosis, stroke, Alzheimer's disease, Parkinson's disease, attention-deficit hyperactivity disorder, migraine, epilepsy, vascular dementia, vascular encephalopathy (with hypertension, diabetes, or dyslipidemia), multiple sclerosis, cerebrovascular insufficiency, brain tumors (glioma, astrocytoma, glioblastoma, and meningioma), cranial nerve neuropathy (facial palsy and trigeminal neuralgia), mental retardation, and posttraumatic brain injury syndrome (Figure 1). The distribution of *APOE* genotypes in the Iberian peninsula is as follows: *APOE-2/2* 0.32%, *APOE-2/3* 7.3%, *APOE-2/4* 1.27%, *APOE-3/3* 71.11%, *APOE-3/4* 18.41%, and *APOE-4/4* 1.59% (Figure 1). There is a clear accumulation of *APOE-4* carriers among patients with AD (*APOE-3/4* 30.30%; *APOE-4/4* 6.06%) (*P* < 0.001) and vascular dementia (*APOE-3/4* 35.85%, *APOE-4/4* 6.57%) (*P* < 0.001) as compared to controls. The distribution and frequencies of *APOE* genotypes in AD also differ from those of patients with anxiety (*P* < 0.001), depression (*P* < 0.001), psychosis (*P* < 0.005), migraine (*P* < 0.03), vascular encephalopathy (*P* < 0.001), and posttraumatic brain injury syndrome (*P* < 0.03). Significant differences are also present between vascular dementia and anxiety (*P* < 0.001), depression (*P* < 0.001), psychosis (*P* < 0.001), migraine (*P* < 0.002), vascular encephalopathy (*P* < 0.001), and posttraumatic brain injury syndrome (*P* < 0.008) (Figure 1). 

 The pharmacogenomics of AD is still in a very primitive stage. In over 100 clinical trials for dementia, *APOE* has been used as the only gene of reference for the pharmacogenomics of AD [5, 7, 9, 105, 112, 113, 138, 139]. Several studies indicate that the presence of the *APOE-4* allele differentially affects the quality and extent of drug responsiveness in AD patients treated with cholinergic enhancers (tacrine, donepezil, galantamine, and rivastigmine), neuroprotective compounds (nootropics), endogenous nucleotides (CDP-choline), immunotrophins (anapsos), neurotrophic factors (cerebrolysin), rosiglitazone, or combination therapies [5–7, 9, 105, 112, 113, 138, 140]; however, controversial results are frequently found due to methodological problems, study design, and patient recruitment in clinical trials.

*APOE-4* carriers show a less significant therapeutic response to tacrine (60%) than patients with no *APOE-4 *[141]. The frequency of *APOE-4 *alleles was higher in responders to a single oral dose of tacrine [142]. About 80% of *APOE-4*(−) AD patients showed marked improvement after 30 weeks of treatment with tacrine, whereas 60% of *APOE-4*(+) carriers had a poor response [141]. Others found no differences after 6 months of treatment with tacrine among *APOE *genotypes, but after 12 months the CIBIC scores revealed that *APOE-4 *carriers had declined more than the *APOE-2 *and *APOE-3 *patients, suggesting that a faster rate of decline was evident in the *APOE-4 *patients, probably reflecting that *APOE-4 *inheritance is a negative predictor of treatment of tacrine in AD [143]. It has also been shown that the *APOE* genotype may influence the biological effect of donepezil on APP metabolism in AD [144]. Prospective studies with galantamine in large samples of patients in Europe [145] and in USA [146] showed no effect of *APOE* genotypes on drug efficacy, but a retrospective study with a small number of AD cases in Croatia showed the intriguing result of 71% responders to galantamine treatment among *APOE-4* homozygotes [147]. MacGowan et al. [148] reported that gender is likely to be a more powerful determinant of outcome of anticholinesterase treatment than *APOE* status in the short term. In contrast, other studies do not support the hypothesis that *APOE* and gender are predictors of the therapeutic response of AD patients to tacrine or donepezil [149, 150]. Petersen et al. [151] showed that *APOE-4* carriers exhibited a better response to donepezil. Similar results have been found by Bizzarro et al. [152]; however, Rigaud et al. [150] did not find any significant difference between *APOE-4*-related responders and nonresponders to donepezil. An *APOE*-related differential response has also been observed in patients treated with other compounds devoid of acetylcholinesterase inhibiting activity (CDP-choline, anapsos) [153, 154] suggesting that *APOE*-associated factors may influence drug activity in the brain either directly acting on neural mechanisms or indirectly influencing diverse metabolic pathways [155].

In long-term open clinical trials with a multifactorial treatment, *APOE-4/4* carriers are the worst responders [5–7, 9, 105, 112, 113]. With a similar therapeutic protocol, *PSEN1-1/1* homozygotes are the worst responders and *PSEN1-2/2* carriers are the best responders [5]. Significant *ACE*-related therapeutic responses to multifactorial treatments have also been reported [5, 6]. Among *ACE*-I/D variants, *ACE*-D/D patients were the worst responders and *ACE*-I/D carriers were the best responders, with *ACE*-I/I showing an intermediate positive response [5, 6]. *ACE*-related biochemical and hemodynamic phenotypes have been studied in patients with AD [4, 9, 97]. *ACE*-I/I patients tend to be younger than *ACE*-I/D or *ACE*-D/D patients at the time of diagnosis and also to show a more severe cognitive deterioration. Serum ApoE, total cholesterol, LDL-cholesterol, HDL-cholesterol, nitric oxide, histamine, and ACE levels are higher in *ACE*-I/I carriers than in patients with the other genotypes; in contrast, serum triglyceride and VLDL levels are notably lower in *ACE*-I/I patients compared to patients harboring the *ACE*-I/D or *ACE*-D/D genotypes, whereas A*β* levels do not show any clear difference among *ACE*-related genotypes. Cerebrovascular function tends to be worse in *ACE*-D/D, with lower brain blood flow velocities and higher pulsatility and resistance indices, than in *ACE*-I/D (intermediate cerebrovascular hemodynamics) or *ACE*-I/I (almost normal cerebrovascular function) [4, 6, 9, 97]. Low triglyceride levels may facilitate cerebrovascular function. *ACE*-I/I patients with the highest cholesterol levels are the worst in mental performance. These data might suggest an association of poor cerebrovascular function with *ACE*-D/D and *ACE*-I/D and an association of alterations in lipid metabolism with *ACE*-I/I [4, 6].

Both *APOE* and *ACE* variants also affect behavior and the modification of behavioral changes (mood and anxiety) in dementia after nonpsychotropic pharmacological treatment [4, 6, 9, 105, 113]. At baseline, all *APOE* variants show similar anxiety and depression rates, except the *APOE-4/4* carriers who differed from the rest in significantly lower rates of anxiety and depression. Remarkable changes in anxiety were found among different *APOE* genotypes. Practically all *APOE* variants responded with a significant diminution of anxiogenic symptoms, except patients with the *APOE-4/4* genotype, who only showed a slight improvement. The best responders were *APOE-2/4* > *APOE-2/3* > *APOE-3/3* > *APOE-3/4* carriers. The potential influence of *APOE* variants on anxiety and cognition in AD does not show a clear parallelism, suggesting that other more complex mechanisms are involved in the onset of anxiety in dementia. Concerning depression, all *APOE *genotypes improved their depressive symptoms with treatment except those with the *APOE-4/4* genotype, which worsen along the treatment period. The best responders were *APOE-2/4* > *APOE-2/3* > *APOE-3/3* > *APOE-3/4*, and the worst responder was *APOE-4/4* [4, 6]. Patients with each one of the 3 *ACE*-I/D indel variants were equally anxiogenic and depressive at baseline and all of them responded favorably to the multifactorial protocol by gradually reducing anxiety and depressive symptoms over the 12-month treatment period. The best responders were *ACE*-I/D > *ACE*-D/D > *ACE*-I/I. Depressive symptoms were also similarly improved in all *ACE*-I/D variants. The best responders were *ACE*-I/D > *ACE*-D/D > *ACE*-I/I. Comparatively, the worst responders among *ACE*-I/D variants were carriers of the *ACE*-I/I genotype, which were also the poorest responders in anxiety and cognition [4, 6, 115].

The combination of *APOE* and *ACE* polymorphic variants in bigenic clusters yielded different anxiety and depression patterns at baseline and after one year of treatment. The most anxiogenic patients at baseline were those with the 23DD, 44ID, and 34II genotypes, and the least anxiogenic patients were those harboring the 23II, 44DD, and 23ID genotypes. The most depressive clusters at baseline were those harboring the 23DD, 33ID, and 33II genotypes, with a clear accumulation of *APOE-3/3* carriers in these groups, and the least depressive clusters were those represented by carriers of the 23II, 44ID, and 23ID genotypes. All bigenic clusters showed a positive anxiolytic and antidepressive response to the multifactorial treatment, except 44DD carriers who exhibited the worst response [4, 6, 115].

*APOE* influences liver function and *CYP2D6*-related enzyme activity probably via regulation of hepatic lipid metabolism. It has been observed that *APOE* may influence liver function and drug metabolism by modifying hepatic steatosis and transaminase activity. There is a clear correlation between *APOE*-related TG levels and GOT, GPT, and GGT activities in AD [4, 6]. Both plasma TG levels and transaminase activity are significantly lower in AD patients harboring the *APOE-4/4* genotype, probably indicating (i) that low TG levels protect against liver steatosis and (ii) that the presence of the *APOE-4* allele influences TG levels, liver steatosis, and transaminase activity. Consequently, it is very likely that *APOE* influences drug metabolism in the liver through different mechanisms, including interactions with enzymes such as transaminases and/or cytochrome P450-related enzymes encoded in genes of the CYP superfamily [4, 6, 115].

When *APOE* and *CYP2D6* genotypes are integrated in bigenic clusters and the *APOE*+*CYP2D6*-related therapeutic response to a combination therapy is analyzed in AD patients, it becomes clear that the presence of the *APOE-4/4* genotype is able to convert pure CYP2D6*1/*1 EMs into full PMs, indicating the existence of a powerful influence of the *APOE-4* homozygous genotype on the drug-metabolizing capacity of pure *CYP2D6*-EMs. In addition, a clear accumulation of *APOE-4/4* genotypes is observed among *CYP2D6* PMs and UMs [5].

From these studies we can conclude the following. (i) Most studies with acetylcholinesterase inhibitors indicate that the presence or absence of the *APOE-4* allele influences the therapeutic outcome in patients with AD. (ii) Multifactorial treatments combining neuroprotectants, endogenous nucleotides, nootropic agents, vasoactive substances, cholinesterase inhibitors, and NMDA antagonists associated with metabolic supplementation on an individual basis adapted to the phenotype of the patient may be useful to improve cognition and to slow down disease progression in AD. (iii) The therapeutic response in AD seems to be genotype-specific under different pharmacogenomic conditions. (iv) In monogenic-related studies, patients harboring the *APOE-4/4* genotype are the worst responders. (v) *APP*, *PSEN1,* and *PSEN2* mutations influence the therapeutic response in AD. (vi) In trigenic-related studies (*APOE *+ *PSEN1 *+ *PSEN2*) the best responders are those patients carrying the 331222-, 341122-, 341222-, and 441112-genomic clusters. (vii) The worst responders in all genomic clusters are patients with the 441122 + genotype. (viii) The interaction of several AD-related genes seems to be determinant for drug efficacy and safety. (ix) *APOE-CYP2D6* interactions might influence the therapeutic response in AD via changes in lipid metabolism and liver function. (x) *APOE* may also interact with *PSEN1*, *ACE*, *A2M,* and other genes to regulate the effect of drugs on cognition and behavioral changes in dementia. (xi) The *APOE-4/4* genotype seems to accelerate neurodegeneration anticipating the onset of the disease by 5–10 years, and, in general, *APOE-4/4* carriers show a faster disease progression and a poorer therapeutic response to all available treatments than any other polymorphic variant. (xii) Pharmacogenomic studies using monogenic, bigenic, trigenic, tetragenic, or polygenic clusters as a harmonization procedure to reduce genomic heterogeneity in clinical trials are very useful in order to widen the therapeutic scope of limited pharmacological resources [4–7, 9, 97, 105–115].

## 7. *APOE*-Related Therapeutic Response to a Multifactorial Therapy in Alzheimer's Disease

**Figure 2:**
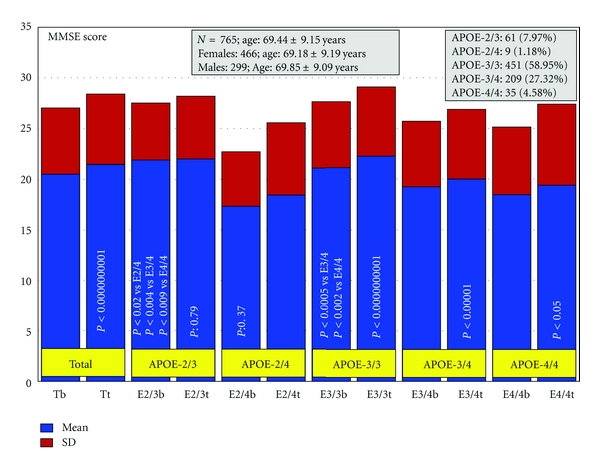
*APOE*-related therapeutic response to a multifactorial therapy in patients with dementia. Cognitive performance (MMSE Score). Tb: basal MMSE score prior to treatment; Tt: MMSE score after 3 months of treatment in the total sample. E2/3b: basal MMSE score in *APOE-2/3 *carriers; E2/3t: MMSE score after treatment in *APOE-2/3 *carriers; E2/4b: Basal MMSE score in *APOE-2/4 *carriers; E2/4t: MMSE score after treatment in *APOE-2/4 *carriers; E3/3b: basal MMSE score in *APOE-3/3 *carriers; E3/3t: MMSE score after treatment in *APOE-3/3* carriers; E3/4b: basal MMSE score in *APOE-3/4* carriers; E3/4t: MMSE score after treatment in* APOE-3/4* carriers; E4/4b: basal MMSE score in *APOE-4/4* carriers; E4/4: MMSE score after treatment in *APOE-4/4* carriers.

Patients with dementia (*N* = 765, age: 69.44 ± 9.15 years, range: 50–96 years; 466 females, age: 69.18 ± 9.19 years, range: 50–96 years; 299 males, age: 69.85 ± 9.09 years, range: 50–91 years; *P* < 0.01) received for three months a multifactorial therapy integrated by CDP-choline (500 mg/day, p.o.), Nicergoline (5 mg/day, p.o.), Sardilipin (E-SAR-94010) (LipoEsar) (250 mg, t.i.d.), and Animon Complex (2 capsules/day), a nutraceutical compound integrated by a purified extract of *Chenopodium quinoa* (250 mg), ferrous sulphate (38.1 mg equivalent to 14 mg of iron), folic acid (200 *μ*g), and vitamin B_12_ (1 *μ*g) per capsule (RGS: 26.06671/C). Patients with chronic deficiency of iron (<35 *μ*g/mL), folic acid (<2.5 ng/mL), or vitamin B_12_ (<150 pg/mL) received an additional supplementation of iron (80 mg/day), folic acid (5 mg/day), and B complex vitamins (B_1_ 15 mg/day; B_2_ 15 mg/day; B_6_ 10 mg/day; B_12_ 10 *μ*g/day; nicotinamide 50 mg/day), respectively, to maintain stable levels of serum iron (50–150 *μ*g/mL), folic acid (5–20 ng/mL), and vitamin B_12_ levels (500–1,000 pg/mL) in order to avoid the negative influence of all these metabolic factors on cognition [6, 114]. Patients with hypertension (>150/85 mmHg) received Enalapril (20 mg/day). The frequency of *APOE *genotypes was *APOE-2/3* 7.97%; *APOE-2/4* 1.18%; *APOE-3/3* 58.95%; *APOE-3/4* 27.32%; and *APOE-4/4* 4.58% (Figure 2). Blood pressure, psychometric assessment (Mini-Mental State Examination (MMSE); ADAS; Hamilton Rating Scale-Depression (HAM-D); Hamilton Rating Scale-Anxiety (HAM-A)), and blood parameters (glucose, total cholesterol, HDL-cholesterol, LDL-cholesterol, triglyceride, iron, folate, vitamin B_12_, and TSH, T_4_) were evaluated at baseline and after 3 months of treatment [42].

 Systolic (*P* < 0.0002) and diastolic blood pressure (*P* < 0.001), cognitive function (as assessed by MMSE, 20.51 ± 6.51 versus 21.45 ± 6.95, *P* < 0.0000000001; ADAS-Cog, 22.94 ± 13.87 versus 21.23 ± 12.84, *P* < 0.0001; ADAS-Non-Cog, 5.26 ± 4.18 versus 4.15 ± 3.63, *P* < 0.0000000001; ADAS-Total, 27.12 ± 16.93 versus 24.28 ± 15.06, *P* < 0.00009), and mood (HAM-A, 11.35 ± 5.44 versus 9.79 ± 4.33, *P* < 0.0000000001; HAM-D, 10.14 ± 5.23 versus 8.59 ± 4.30, *P* < 0.0000000001) improved after treatment. Glucose levels did not change. Total cholesterol levels (224.78 ± 45.53 versus 203.64 ± 39.69  mg/dL, *P* < 0.0000000001), HDL-cholesterol levels (54.11 ± 14.54 versus 52.54 ± 14.86 mg/dL, *P* < 0.0001), and LDL-cholesterol levels (148.15 ± 39.13 versus 128.89 ± 34.83 mg/dL, *P* < 0.0000000001) were significantly reduced, whereas triglyceride levels increased (111.99 ± 67.14 versus 120.69 ± 67.14 mg/dL, *P* < 0.0006) after 3 months of combined treatment. Folate (7.07 ± 3.61 versus 18.14 ± 4.23 ng/mL, *P* < 0.000000001) and vitamin B_12_ levels (459.65 ± 205.80 versus 689.78 ± 338.82 pg/mL, *P* < 0.000000001) also increased, and both TSH and T_4_ levels remained unchanged after treatment. The response rate in terms of cognitive improvement was as follows: 59.74% responders (RRs), 24.44% nonresponders (NRs), and 15.82% stable responders (SRs) (no change in MMSE score after three months of treatment). The response rate in cholesterol levels was very similar: 57.78% RRs, 28.50% NRs, and 13.72% SRs [42].

In this study, the basal MMSE score differed in *APOE-2/3 *carriers with respect to *APOE-2/4* (*P* < 0.02), *APOE-3/4* (*P* < 0.004), and *APOE-4/4* carriers (*P* < 0.0009), in *APOE-3/3 *versus* APOE-3/4* (*P* < 0.0005), and *APOE-3/3* versus *APOE-4/4 *(*P* < 0.002). The best responders were *APOE-3/3* (*P* < 0.0000000001) > *APOE-3/4* (*P* < 0.00001) > *APOE-4/4 *carriers (*P* < 0.05) (Figure 2). Patients harboring the *APOE-2/3* and *APOE-2/4 *genotypes did not show any significant improvement. The response rate by genotype was the following: *APOE-2/3*: 44.26% RRs, 36.07% NRs, and 19.67% SRs; *APOE-2/4*: 55.56% RRs, 44.44% NRs, and 0.0% SRs; *APOE-3/3*: 63.42% RRs, 21.06% NRs, and 15.52% SRs; *APOE-3/4*: 56.94% RRs, 27.75% NRs, and 15.31% SRs; *APOE-4/4*: 51.43% RRs, 28.57% NRs, and 20.00% SRs [42].

**Figure 3:**
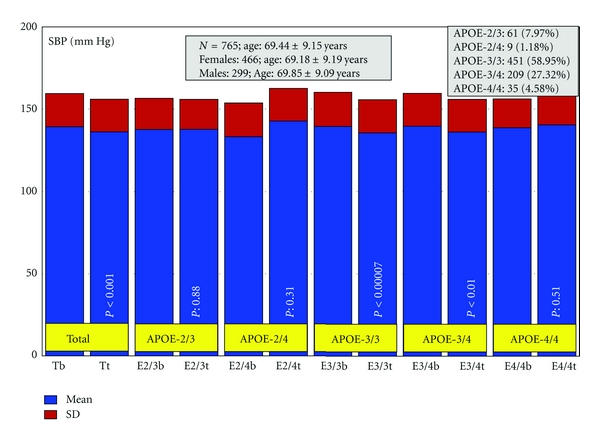
*APOE*-related systolic blood pressure response to a multifactorial therapy in patients with dementia. Tb: basal systolic blood pressure (SBP) prior to treatment; Tt: SBP after 3 months of treatment in the total sample. E2/3b: basal SBP in *APOE-2/3 *carriers; E2/3t: SBP after treatment in *APOE-2/3 *carriers; E2/4b: basal SBP in *APOE-2/4 *carriers; E2/4t: SBP after treatment in *APOE-2/4 *carriers; E3/3b: basal SBP in *APOE-3/3 *carriers; E3/3t: SBP after treatment in *APOE-3/3* carriers; E3/4b: basal SBP in *APOE-3/4* carriers; E3/4t: SBP after treatment in* APOE-3/4* carriers; E4/4b: basal SBP in *APOE-4/4* carriers; E4/4: SBP after treatment in *APOE-4/4* carriers.

Systolic blood pressure (SBP) was significantly reduced in patients with the *APOE-3/3* (*P* < 0.00007) and *APOE-3/4 *genotypes (*P* < 0.01), and diastolic blood pressure exhibited a similar pattern (*APOE-3/3*, *P*  < 0.005; *APOE-3/4*, *P* < 0.01), with no changes in either SBP or DBP in *APOE-2/3*, *APOE-2/4, *and *APOE-4/4 *carriers (Figure 3) [42].

 Glucose levels tended to decrease in *APOE-4* allele carriers, but only patients with the *APOE-3/4* genotype showed a significant reduction in glucose levels (*P* < 0.02). In contrast, *APOE-2/3* carriers showed a tendency to increased glucose levels [42].

## 8. *APOE*-Related Blood Lipid Response to Sardilipin (E-SAR-94010)

**Figure 4:**
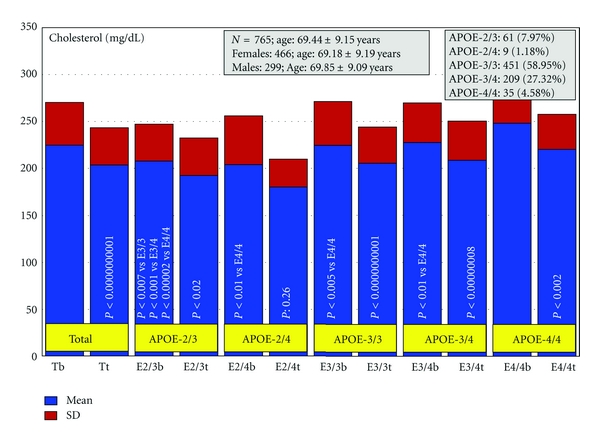
*APOE*-related changes in total cholesterol levels in patients with dementia treated with E-SAR-94010. Tb: basal total cholesterol (CHO) levels prior to treatment; Tt: CHO levels after 3 months of treatment in the total sample. E2/3b: basal CHO levels in *APOE-2/3 *carriers; E2/3t: CHO levels after treatment in *APOE-2/3 *carriers; E2/4b: basal CHO levels in *APOE-2/4 *carriers; E2/4t: CHO levels after treatment in *APOE-2/4 *carriers; E3/3b: basal CHO levels in *APOE-3/3 *carriers; E3/3t: CHO levels after treatment in *APOE-3/3* carriers; E3/4b: basal CHO levels in *APOE-3/4* carriers; E3/4t: CHO levels after treatment in* APOE-3/4* carriers; E4/4b: basal CHO levels in *APOE-4/4* carriers; E4/4: CHO levels after treatment in *APOE-4/4* carriers.

Basal cholesterol levels were significantly different in patients with the *APOE-2/3 *genotype versus *APOE-3/3 *(*P* < 0.007), versus* APOE-3/4 *(*P* < 0.001), versus* APOE-4/4* (*P* < 0.00002); *APOE-2/4 *versus* APOE-4/4 *(*P* < 0.01); *APOE-3/3 *versus* APOE-4/4 *(*P* < 0.005 ); *APOE-3/4* versus* APOE-4/4* (*P* < 0.01) (Figure 4) [42].

 The highest cholesterol levels were seen in *APOE-4/4 *> *APOE-3/4* > *APOE-3/3.* All patients showed a clear reduction in cholesterol levels after treatment with Sardilipin. This was particularly significant in *APOE-3/3* (*P* < 0.0000000001) > *APOE-3/4* (*P* < 0.00000008) > *APOE-4/4 *(*P* < 0.002) > *APOE-2/3* (*P* < 0.02) > *APOE-2/4 *carriers (*P*: 0.26) (Figure 4). The response rate by genotype was as follows: *APOE-2/3*: 63.93% RRs, 29.51% NRs, and 6.56% SRs; *APOE-2/4*: 44.44% RRs, 22.22% NRs, and 33.34% SRs; *APOE-3/3*: 54.32% RRs, 28.16% NRs, and 17.52% SRs; *APOE-3/4*: 53.59% RRs, 31.58% NRs, and 14.83% SRs; *APOE-4/4*: 65.71% RRs, 20.00% NRs, and 14.29% SRs [42].

**Figure 5:**
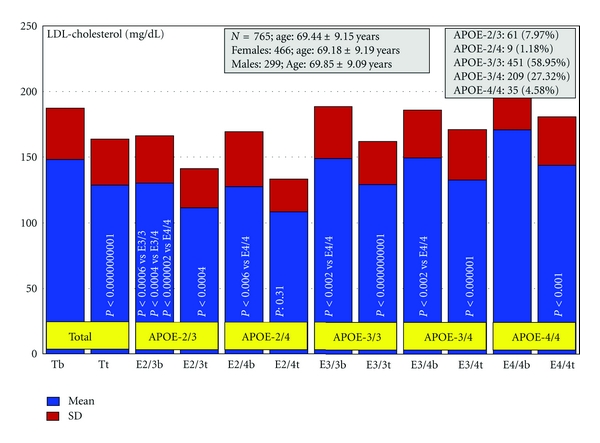
*APOE*-related changes in LDL-cholesterol levels in patients with dementia treated with E-SAR-94010. Tb: basal LDL-cholesterol (LDL-CHO) levels prior to treatment; Tt: LDL-CHO levels after 3 months of treatment in the total sample. E2/3b: basal LDL-CHO levels in *APOE-2/3 *carriers; E2/3t: LDL-CHO levels after treatment in *APOE-2/3 *carriers; E2/4b: basal LDL-CHO levels in *APOE-2/4 *carriers; E2/4t: LDL-CHO levels after treatment in *APOE-2/4 *carriers; E3/3b: basal LDL-CHO levels in *APOE-3/3 *carriers; E3/3t: LDL-CHO levels after treatment in *APOE-3/3* carriers; E3/4b: basal LDL-CHO levels in *APOE-3/4* carriers; E3/4t: LDL-CHO levels after treatment in* APOE-3/4* carriers; E4/4b: basal LDL-CHO levels in *APOE-4/4* carriers; E4/4: LDL-CHO levels after treatment in *APOE-4/4* carriers.

HDL-cholesterol levels significantly decreased in *APOE-3/3* (*P* < 0.001) > *APOE-3/4 *(*P* < 0.05), with no significant changes in patients with other genotypes. In contrast, LDL-cholesterol levels showed identical changes to those observed in total cholesterol, with similar differences among genotypes at baseline and almost identical decreased levels after treatment (*APOE-3/3*, *P* > 0.0000000001; >*APOE-3/4*, *P* < 0.00001; >*APOE-2/3*, *P* < 0.0004; >*APOE-4/4*, *P* < 0.001; >*APOE-2/4*, *P*: 0.31) (Figure 5) [42].

 Paradoxically, triglyceride levels tended to increase in all *APOE* genotypes (*APOE-3/3*, *P*  < 0.01; >*APOE-4/4*, *P* < 0.03; >*APOE-2/3*, *P*: 0.12; >*APOE-3/4*, *P*: 0.17), except in *APOE-2/4* carriers, who showed a tendency to decrease. Basal triglyceride levels were significantly lower in *APOE-4/4* carriers than in *APOE-2/3* (*P* < 0.03) and *APOE-3/4* carriers (*P* < 0.04) [42].

Sardilipin (E-SAR-94010, LipoEsar, LipoSea) is a natural product extracted from the marine species *Sardina pilchardus*, by means of nondenaturing biotechnological procedures [156]. The main chemical compounds of LipoEsar are lipoproteins (60–80%) whose micelle structure probably mimics that of physiological lipoproteins involved in lipid metabolism. In preclinical studies, sardilipin has shown to be effective in (i) reducing blood cholesterol (CHO), triglyceride (TG), uric acid (UA), and glucose (Glu) levels, as well as liver alanine aminotransferase (ALT) and aspartate aminotransferase (AST) activity, (ii) enhancing immunological function by regulating both lymphocyte and microglia activity, (iii) inducing antioxidant effects mediated by superoxide dismutase activity, and (iv) improving cognitive function [97, 156, 157].

According to these results, it appears that the therapeutic response of patients with dyslipidemia to sardilipin is *APOE*-related. The best responders were patients with *APOE-3/3 *> *APOE-3/4 *> *APOE-4/4*. Patients with the other *APOE *genotypes (*2/2*,* 2/3*, and *2/4*) did not show any hypolipemic response to this novel compound [97, 157]. In patients with dementia, the effects of sardilipin were very similar to those observed in patients with chronic dyslipidemia, suggesting that the lipid-lowering properties of sardilipin are *APOE*-dependent.

Clinical studies have revealed that sardilipin reduces blood total cholesterol (T-CHO) (20–30%), Glu (5–10%), UA (10–15%), TG (30–50%), ALT, and AST, after 1–3 months of treatment at a daily dose of 250–500 mg (t.i.d). The effect on T-CHO is the result of decreasing LDL-CHO levels and increasing HDL-CHO levels in parallel with an improvement in hepatic protection reflected by reduction in ALT, AST, and GGT activity, as the result of reducing liver steatosis. Both LDL and HDL levels are modulated by dietary, behavioral, and genetic factors [97]. Most of these therapeutic effects on the regulation of lipid metabolism tend to show an age-dependent pattern and are also associated with specific genomic profiles in the population. In addition, sardilipin diminishes the size of xanthelasma plaques by 30–60% after 6–9 months of treatment and specifically protects against the hepatotoxicity induced by statins. Similar effects can be observed on atheromatous plaques on the aortic wall of patients with familial and sporadic dyslipidemia/hyperlipidemia. The daily administration of 1,000–1,500 mg/day of E-SAR p.o. for three months tends to reduce the average size of atherosclerotic plaques on the aortic wall by 10%. This effect is more significant in patients harboring the *APOE-3/3* than in *APOE-3/4* carriers in whom the size of the plaque is approximately 30–40% larger than in *APOE-3/3 *carriers [97, 158].

## 9. New Insights into *APOE*-Related Pathogenesis and Therapeutics in Alzheimer's Disease

*APOE* is a pleiotropic gene with many polymorphic activities, most of them influencing AD pathogenesis. In this regard, the influence of *APOE* variants on AD therapeutics cannot be neglected, especially taking into account that (a) *APOE* polymorphic variants by themselves are enough to modify the therapeutic response to conventional antidementia drugs, (b) ApoE interacts with many receptors and participates in a large number of metabolic cascades and signaling pathways, and (c) the presence of the *APOE-4* allele can alter the phenotypic profile of *CYP2D6* genotype-related drug metabolizers and probably also of other cytochrome P450 enzymes, such as those encoded by the *CYP3A5* gene, which affect more than 50% of the drugs currently prescribed in the clinical setting. Moreover, many metabolic pathways in which *APOE* participates (e.g., lipid metabolism, APP/A*β* processing, cardiovascular and cerebrovascular function, etc.) are involved in pathogenic processes that represent major risk factors of dementia (e.g., atherosclerosis, hypercholesterolemia, hypertension, and brain hypoperfusion), which can be potentially predictable and preventable with therapeutic intervention.

ApoE is a ligand for the 7 identified mammalian members of the evolutionarily conserved low-density lipoprotein (LDL) receptor family: the low-density lipoprotein receptor (LDLR), ApoE receptor 2 (ApoER2), the very-low-density lipoprotein receptor (VLDLR), multiple epidermal growth factor (EGF) repeat-containing protein (MEGF7), megalin, LDL-related-protein-1 (LRP1) and LDL-related protein-1b (LRP1b) [159]. The LDLR family consists of over 10 receptors that function in receptor-mediated endocytosis and cellular signaling. Together with LDLR itself, the family includes LRP/LRP1, megalin/LRP2, VLDLR, ApoER2/LRP8, SORLA-1/LR11, LRP4, LRP5, LRP6, and LRP1B. Most of the ApoE receptors have been found in the CNS where they participate in endocytosis, intracellular signaling, synaptic plasticity, and A*β* metabolism [159]. ApoE receptors have been suggested to act as clearance mechanisms for A*β* and have also been implicated in the production of A*β*. LRP interacts with APP through the intracellular adaptor protein FE65 or via direct binding to the KPI domain, and its endocytosis facilitates APP endocytic trafficking and A*β* production [160–162]. SORLA/LR11 alters APP trafficking and APP processing by *β*- and *γ*-secretases [163–165]. It has also been suggested that soluble ApoE receptors could play roles as dominant negative regulators of ApoE, and thus understanding their generation and actions might be important for understanding normal and pathological functions of ApoE in the CNS and in AD [159].

It might be possible that normalization of biological parameters associated with *APOE*-related pathogenic pathways contributing to brain dysfunction and neurodegeneration could be beneficial in terms of prevention and/or slowing the clinical course of dementia. In this strategic category we can include the following: (i) lipid metabolism dyshomeostasis, (ii) *APOE*-related APP/A*β* processing, (iii) blood pressure control, (iv) atherogenesis, (v) cerebrovascular hemodynamics, and (vi) neuroprotection.

Cardiovascular and cerebrovascular disorders associated with lipid metabolism disturbance and atherosclerosis represent major risk factors for dementia [119, 120, 166]. Atherosclerosis is the primary cause of heart disease and stroke in which genetic and environmental factors converge [167]. More than 90% of patients with dementia older than 70–80 years of age show signs of atherosclerosis in their arteries and a clear cerebrovascular component in their dementia process. It is very likely that pure AD is practically absent in octogenarians in whom the prevalent diagnosis is vascular or mixed-dementia [119, 120, 166] in which the *APOE-4* allele also accumulates [97, 110, 111, 168].

*APOE* genotypes directly influence lipid metabolism and atherosclerosis. The presence of the *APOE-4* allele contributes to the phenotypic manifestation of atherosclerosis, brain amyloid angiopathy, and cerebral white matter damage [169]. The size of atheroma plaques in the abdominal and thoracic aorta of patients with dementia and/or dyslipidemia is significantly larger in *APOE-4* carriers than in *APOE-3* carriers [97, 110, 111]. In addition, the effect of lipid-lowering agents on atheroma plaques is *APOE* related with a more effective response in *APOE-3* carriers [97, 110, 111].

Evidence from epidemiological, *in vitro*, and *in vivo* studies suggests that brain cholesterol may play a role in AD. The exact nature and magnitude of this role is unknown, but a number of possibilities have emerged, including modulation of APP cleavage pathways and A*β* production and clearance, APOE-mediated cholesterol transport, and cholesterol efflux from the brain [170–172].

Cholesterol is implicated in the production of A*β*, the primary constituent of senile plaques in the AD brain [173–177]. In *APP* transgenic mice, hypercholesterolemia correlates with increased A*β* levels and more severe amyloid plaque load [178, 179]. Some retrospective epidemiological studies indicate that statin therapy might decrease AD risk [180], but statins do not alter serum A*β* levels [181, 182] and in some cases may worsen cognitive function, increase brain A*β* load, and/or activate inflammatory responses involving microglia [120, 166, 183]. Some studies have reported an association between high cholesterol levels and AD risk [184] and increased brain A*β*1–42 levels [185]. Defective binding of ApoE to heparan sulfate proteoglycans (HSPGs) is associated with increased risk of atherosclerosis and AD probably due to the inefficient clearance of lipoprotein remnants from the liver with negative consequences for neuronal repair [186]. CYP46*C (cholesterol 24-hydroxylase) along with *APOE-4* was found associated with higher cognitive decline in AD and both variants synergistically increase the risk of AD [187–189] as well as brain and CSF A*β* load [190]. Deficiency of the cholesterol transporter ABCA1 produced by glial cells impairs ApoE metabolism in the CNS [191]. Some studies also indicate that genetic variants of *ABCA1* modify AD risk and tau- and A*β*-related pathogenesis [192]; however, other studies have demonstrated that several SNPs in the multi-drug resistance (*ABCB1*) gene (*MRD1*) (C1236T in exon 12, G2677T/A in exon 21, and C3435T in exon 26) do not show association with AD [193]; in contrast, *ABCA2* has been reported to be a strong genetic risk factor for early-onset AD [194, 195]. The ATP-binding cassette (ABC) superfamily consists of membrane proteins that transport a wide variety of substrates across membranes [196]. *ABCA1* and *ABCG1* play a pivotal role in the regulation of neuronal cholesterol to ApoE discs and in suppression of APP processing to generate A*β*. ABCA1 is required for normal brain ApoE levels and for lipidation of astrocyte-secreted ApoE [197], and the absence of ABCA1 decreases soluble ApoE levels but does not diminish A*β* deposition in AD murine models [198], whereas others have reported increased A*β* deposition in *APP23* and *PDAPP* mice in the absence of ABCA1, suggesting that despite substantially lower ApoE levels, poorly lipidated ApoE produced in the absence of ABCA1 is strongly amyloidogenic [199, 200]. ABCA1 protein expression is induced by ligands of the nuclear hormone receptors of the retinoid X receptor and liver X receptor family. Treatment of neuroblastoma cells with retinoic acid and 22(R)-hydroxycholesterol causes significant increases in secreted A*β*40 and A*β*42, and treatment with a nonsteroidal liver X receptor ligand, TO-901317, similarly increases A*β*40 and A*β*42 levels, which can be reduced by RNAi blocking of ABCA1 expression [201]. Maintenance of an adequate supply of cholesterol is important for neuronal function whereas excess cholesterol promotes APP cleavage and generation of toxic A*β* isoforms [202]. Impaired recycling of APOE-4 is associated with intracellular cholesterol accumulation [203]. Cholesterol- and sphingolipid-rich membrane microdomains are involved in regulating trafficking and processing of APP. In this metabolic pathway, the amyloidogenic processing of APP depends on lipid rafts since access of *α*- and *β*-secretase to APP may be determined by dynamic interactions of APP with membrane lipid microdomains [204]. *γ*-Secretase is also located in lipid raft microdomains of post-Golgi and endosomes that are implicated in APP processing [205]. Methyl-*β*-cyclodextrin and leptin reduce *β*-secretase activity in neuronal cells possibly by altering the lipid composition of membrane lipid rafts. This phenotype contrasts treatments with cholesterol and etomoxir, an inhibitor of carnitine-palmitoyl-transferase-1. Conversely, inhibitors of acetyl CoA carboxylase and fatty acid synthase mimic the action of leptin. Leptin is also able to increase ApoE-dependent A*β* uptake *in vitro*; thus, leptin can modulate bidirectional A*β* kinesis, reducing its levels extracellularly. The chronic administration of leptin to AD-transgenic animals notably reduces the brain A*β* load [206].

*APOE-4* may affect AD risk by conferring high cholesterol levels and thereby increasing A*β* production [207]. *APOE-4* carriers with AD have increased levels of brain and cerebrospinal fluid A*β* and have more extensive plaque pathology [208, 209]; however, with a genomic-based approach, by using an *APOE* knock-in mouse, which expresses each human allele under the endogenous regulatory elements, on a defined C57BL6/J background, Mann et al. [207] were able to demonstrate that the presence of *APOE* significantly increases brain A*β* levels, irrespective of genotype, this indicating an independent role for *APOE* in cholesterol metabolism in the periphery relative to the CNS. In humans, probably thousands of genes may regulate lipid metabolism. Some relevant genes, such as *APOE* (50%), *CETP* (28%), *LIPC* (9%), *APOB* (8%), and *LDLR* (5%) may influence variation in LDL, and *LIPC* (53%), *CETP* (25%), *ABCA1* (10%), *LPL* (6%), and *LDLR* (6%) may influence the HDL variance [210]. The *APOE-2* allele is associated with the lower and the *APOE-4* allele with the higher total plasma cholesterol and LDL-cholesterol levels compared with the *APOE-3* allele [211]. Individuals with *APOE-2* and *APOE-3* reduce plasma cholesterol and LDL-cholesterol levels more than *APOE-4* individuals treated with hydroxymethylglutaryl-CoA (HMG-CoA) reductase inhibitors (statins), gemfibrozil and cholestyramine. In contrast, *APOE-4* carriers might respond better than carriers of other genotypes to probucol. Perimenopausal women with *APOE-2* or *APOE-3* genotypes appear to improve plasma lipoprotein-lipid profiles more than *APOE-4* women under protocols with hormone replacement therapy. Likewise, *APOE-2* and *APOE-3* individuals tend to improve plasma lipid profiles with exercise training more than *APOE-4* individuals [212]. In an attempt to reverse the ApoE deficit in AD, Poirier [213] has reported the identification and characterization of several ApoE inducer agents using a low-throughput screening assay. The old cholesterol-lowering drug, probucol, led to significant increases in CSF ApoE levels and a decrease of CSF A*β*1-42 with effect on CSF tau or lipid peroxide levels [213]. In a prospective, dose-finding, 36-week treatment trial with statins (simvastatin or atorvastatin) conducted in 39 patients with hypercholesterolemia, A*β* levels remained unchanged [214]. The Heart Protection Study Collaborative Group [215] and the Prospective Study of Pravastatin in the Elderly at Risk (PROSPER) [216] have both reported that neither simvastatin nor pravastatin appeared to slow cognitive decline in the elderly during 5 years of treatment in the Heart Protection Study and 3.2 years in the PROSPER.

Since ApoE can protect against cardiovascular disease (e.g., coronary artery disease) via hepatic removal of atherogenic remnant proteins, sequestration of cholesterol from vessel walls, and local antioxidant, antiplatelet, and anti-inflammatory actions, it has been postulated that *APOE* gene transfer might ameliorate a hyperlipidemic profile and exert a beneficial effect at lesion sites to prevent or regress atherosclerosis [217]. Using plasmid vectors expressing allelic human ApoE-2 or ApoE-3 isoforms, Athanasopoulos et al. [217] demonstrated that skeletal muscle was an effective secretory platform for *ApoE* gene augmentation and that muscle-based expression of ApoE-2 after intramuscular plasmid injection in *ApoE*−/− mice was able to reduce atherosclerotic lesions in proximal aorta by 20–30%, with total abolishment of gross dorsal xanthoma (>5 mm diameter) up to 9 months following a single ApoE-2 plasmid administration. The same group of George Dickson [218], 2 years later, with an improved technology, observed an acute regression of advanced and retardation of early aortic atheroma in immunocompetent *ApoE*-deficient mice by administration of a second-generation (E1-, E3-, polymerase-) adenovirus vector expressing human ApoE. Intramuscular injections resulted in low expression of ApoE and afforded no sustainable protection against atherogenesis; in contrast, intravenous (liver-directed) injections into *ApoE*−/− mice resulted in increased plasma ApoE levels accompanied by reductions in plasma cholesterol and normalization of lipoprotein profiles. Liver-directed *ApoE* gene transfer to these mice retarded progression of atherosclerosis by 38% during the 70-day study period, with a progressive decline in ApoE levels and no evoked humoral immune response [218].

## 10. *CYP2D6*-Related Pharmacogenetics

*CYP2D6* is a 4.38 kb gene with 9 exons mapped on 22q13.2. Four RNA transcripts of 1190–1684 bp are expressed in the brain, liver, spleen, and reproductive system where 4 major proteins are identified: *CYP2D6-001*, 55.73 kDa, 497 aa; *CYP2D6-002*, 50.02 kDa, 446 aa; *CYP2D6-004*, 55.19 kDa, 494 aa; *CYP2D6-201*, 48.92 kDa, 493 aa; *CYP2D6-202*, 48.92 kDa, 439 aa; *CYP2D6–203*, 49.65 kDa, 443 aa. This protein is a transport enzyme of the cytochrome P450 subfamily IID or multigenic cytochrome P450 superfamily of mixed-function monooxygenases. The cytochrome P450 proteins are monooxygenases that catalyze many reactions involved in drug metabolism and synthesis of cholesterol, steroids, and other lipids. This protein localizes to the endoplasmic reticulum and is known to metabolize as many as 25% of commonly prescribed drugs and over 60% of current psychotropics. Its substrates include debrisoquine, an adrenergic-blocking drug, sparteine and propafenone, both antiarrhythmic drugs, and amitriptyline, an antidepressant. The gene is highly polymorphic in the population; certain alleles result in the poor metabolizer phenotype, characterized by a decreased ability to metabolize the enzyme substrates. The gene is located near 3 cytochrome P450 pseudogenes on chromosome 22q13.1. In humans there are 3 *CYP2D* pseudogenes: *CYP2D8P1*, *CYP2D8P2,* and *CYP2D7P1*. A frameshift mutation 138delT generates an open reading frame in the pseudogene, cytochrome P4502D7 (*CYP2D7*), and an alternate spliced functional transcript of *CYP2D7* containing partial inclusion of intron 6 was identified in human brain but not in liver or kidney from the same individual. mRNA and protein of the brain variant *CYP2D7* were detected in 6 of 12 human autopsy brains. Genotyping revealed the presence of the frameshift mutation 138delT only in those human subjects who expressed the brain variant *CYP2D7*. Genomic DNA analysis in normal volunteers revealed the presence of functional *CYP2D7* in 4 of 8 individuals [219]. In the liver, the major organ involved in drug metabolism, a minor metabolic pathway mediated by *CYP2D6* metabolizes codeine (prodrug) to morphine (active drug), whereas norcodeine is the major metabolite. In contrast, when expressed in Neuro2a cells, brain variant *CYP2D7* metabolized codeine to morphine with greater efficiency compared with the corresponding activity in cells expressing *CYP2D6*. Morphine binds to *μ*-opioid receptors in certain regions of the CNS, such as periaqueductal gray, and produces pain relief. The brain variant *CYP2D7* and *μ*-opioid receptor colocalize in neurons of the periaqueductal gray area in human brain, indicating that metabolism of codeine to morphine could occur at the site of opioid action. Tissue-specific isoforms of P450 generated by alternate splicing, which mediate selective metabolism of prodrugs within tissues, particularly the brain, to generate active drugs may play an important role in drug action and provide newer insights into the genetics of metabolism [219].

The hepatic cytochrome P450 system is responsible for the first phase in the metabolism and elimination of numerous endogenous and exogenous molecules and ingested chemicals. P450 enzymes convert these substances into electrophilic intermediates, which are then conjugated by phase II enzymes (e.g., UDP glucuronosyltransferases and N-acetyltransferases) to hydrophilic derivatives that can be excreted.

The CYP2D6 enzyme is responsible for metabolizing approximately 25% of pharmaceutical agents. According to the database of the World Guide for Drug Use and Pharmacogenomics [220], 982 drugs are CYP2D6 related: 371 drugs are substrates, over 375 drugs are inhibitors, and 18 drugs are CYP2D6 inducers [220]. In a study to investigate the elimination routes for the 200 drugs most often sold by prescription count in the United States, the majority (78%) of the hepatically cleared drugs were found to be subject to oxidative metabolism via cytochromes P450 of the families 1, 2, and 3, with major contributions from *CYP3A4/5* (37% of drugs) followed by *CYP2C9* (17%), *CYP2D6* (15%), *CYP2C19* (10%), *CYP1A2* (9%), *CYP2C8* (6%), and *CYP2B6* (4%). Clinically well-established polymorphic CYPs (i.e., *CYP2C9*, *CYP2C19*, and *CYP2D6*) were involved in the metabolism of approximately half of those drugs, including (in particular) NSAIDs metabolized mainly by *CYP2C9*, proton-pump inhibitors metabolized by *CYP2C19*, and *β*-blockers and several antipsychotics and antidepressants metabolized by *CYP2D6* [221].

CYP2D6 is one of the most important enzymes catalyzing biotransformation of xenobiotics in the human liver. This enzyme activity shows a high degree of interindividual variability caused in part by its genetic polymorphism, the so-called debrisoquine/sparteine polymorphism. There are 141 *CYP2D6* allelic variants of which −100C>T, −1023C>T, −1659G>A, −1707delT, −1846G>A, −2549delA, −2613–2615delAGA, −2850C>T, −2988G>A, and −3183G>A represent the 10 most important variants [24, 220, 222, 223].

The genetic component influencing *CYP2D6* activity can be determined by genotyping. However, genotyping alone is not sufficient to accurately predict an individual actual *CYP2D6* activity, as this is also influenced by other factors. To determine the exact actual enzymatic activity (phenotyping), adequate probe drugs have to be administered prior to measurements of these compounds and/or their metabolites in body fluids. The enzymatic activity is reflected by various pharmacokinetic metrics such as the partial clearance of a parent compound to the respective *CYP2D6*-mediated metabolite or metabolic ratios [224].

The relative catalytic activities (enzyme kinetics) of three functionally active human *CYP2D6* allelic variants, *CYP2D6.1*, *CYP2D6.10*, and *CYP2D6.17*, were systematically investigated for their ability to metabolize a structurally diverse set of clinically important CYP2D6-metabolized drugs (atomoxetine, bufuralol, codeine, debrisoquine, dextromethorphan, S-fluoxetine, nortriptyline, and tramadol) and the effects of various CYP2D6 inhibitors (cocaine, S-fluoxetine, S-norfluoxetine, imipramine, quinidine, and thioridazine) on these three variants. The most significant difference observed was a consistent but substrate-dependent decrease in the catalytic efficiencies of cDNA-expressed *CYP2D6.10* and *CYP2D6.17* compared with *CYP2D6.1*, yielding 1.32 to 27.9 and 7.33 to 80.4% of the efficiency of *CYP2D6.1*, respectively [225].

## 11. Selected Variants with Clinical Relevance

The *CYP2D6* locus is highly polymorphic, with different *CYP2D6* alleles identified in the general population showing deficient (PM), normal (EM), intermediate (IM), or increased enzymatic activity (UM) [222, 223]. Most individuals (>80%) are EMs; however, remarkable interethnic differences exist in the frequency of the PM and UM phenotypes among different societies all over the world [97, 226, 227]. On average, approximately 6.28% of the world population belongs to the PM category. Europeans (7.86%), Polynesians (7.27%), and Africans (6.73%) exhibit the highest rate of PMs, whereas Orientals (0.94%) show the lowest rate. The frequency of PMs among Middle Eastern populations, Asians, and Americans is in the range of 2-3%. *CYP2D6* gene duplications are relatively infrequent among Northern Europeans, but in East Africa the frequency of alleles with duplication of *CYP2D6* is as high as 29% [228].

The most important SNPs with clinical relevance include the following.

**rs1135840:**

*CYP2D6*-S486T. This SNP is found in the reduced function of the CYP2D6*10, *17, and *41 haplotypes with an allelic frequency of C: 0.635 and G: 0.365.

**rs59421388:**

*CYP2D6*-3183G>A and 3271G>A. This variant is part of the reduced functioning haplotype CYP2D6*29, which is found at an estimated allele frequency of 20% in African Tanzanians.

**rs28371725:**

CYP2D6*41 and *CYP2D6-*2988G>A. *CYP2D6* 2988G>A is an intronic polymorphism that has been shown to be associated with aberrant splicing of *CYP2D6*. This splicing defect leads to the omission of exon 6 from some of the transcribed RNA and leads to a reduction in activity. *CYP2D6* 2988G>A is diagnostic of the haplotype CYP2D6*41, which is believed to be responsible for the IM phenotype in the majority of Caucasians.

**rs16947:**

*CYP2D6*-2850C>T (also named 2938C>T). This is a common SNP in *CYP2D6* and is found in the CYP2D6*2 haplotype among others. CYP2A6*2 activity is slightly reduced but is considered to be in the same extensive metabolizer (EM) category as CYP2D6*1. The presence of *CYP2D6* 2850C>T and the absence of several others is diagnostic of the CYP2D6*2 haplotype.

**rs28371720:**

CYP2D6*9-CYP2D6: 2613–2615delAGA (2701-2703 delAGA). Causes deletion of amino acid K281.

**rs4986774:**

CYP2D6*3 and *CYP2D6-*2549delA (also known as 2637delA in the literature). This causes a frameshift mutation that results in a truncated, nonfunctional protein with an Arg/Gly translation.

**rs3892097:**

CYP2D6*4 and *CYP2D6*-1846G>A. *CYP2D6* 1846G>A (1934G>A) is part of the nonfunctional CYP2D6*4 haplotype. This causes a splicing defect that results in a nonfunctional protein. This variant is responsible for the majority of the PMs found in Caucasian populations, and is also found at much lower frequencies in other populations such as Koreans.

**rs5030655:**

CYP2D6*6 and *CYP2D6-*1707delT. This variant (*CYP2D6-*1707delT or 1795delT) causes a frameshift mutation (Trp/Gly) that results in a truncated, nonfunctional version of *CYP2D6*. This is the defining SNP for CYP2D6*6. Individuals with CYP2D6 *6/*4, *5/*4, or *6/*6 genotypes are poor metabolizers of venlafaxine and are more prone to drug-induced side effects such as nausea, vomiting, and diarrhea. However, *CYP2D6* genotype does not seem to influence venlafaxine efficacy.

**rs61736512:**

*CYP2D6-*1659G>A and 1747G>A. This variant (*CYP2D6 *1659G>A or 1747G>A) is rare in Caucasians and is part of the reduced functioning haplotype CYP2D6*29, which is found at an estimated allele frequency of 20% in African Tanzanians.

**rs28371706:**

*CYP2D6*-1023C>T. *CYP2D6-*1023C>T (1111C>T) was first identified when screening for reduced function alleles in a Zimbabwean population. It was identified as being part of the reduced function haplotype CYP2D6*17 in the African Bantu populations. The presence of *CYP2D6* 1023C>T (1111C>T) and 2850C>T (2938C>T) is diagnostic for CYP2D6*17. *CYP2D6* 1023C>T single mutation exhibited normal function in transfected COS-1 cells, but when made in combination with another mutation led to an increased *K*_*m*_ (decreased affinity) for bufuralol; when the substrate was codeine, *CYP2D6* 1023C>T alone was sufficient to cause an increase in the *K*_*m*_ of *CYP2D6* for codeine, suggesting that this mutation exhibits substrate-specific effects and may contribute to the reduction in function of CYP2D6*17.

**rs1065852:**

*CYP2D6*-100C>T. *CYP2D6* 100C>T (188C>T) is part of both the nonfunctional CYP2D6*4 haplotype and the reduced function CYP2D6 *10 haplotype. Since *CYP2D6* 100C>T is present in both a nonfunctional and a reduced function haplotype, it is not likely to be the causative SNP for the lack of function observed with CYP2D6*4. The presence of *CYP2D6* 100C>T (188C>T) and the absence of *CYP2D6* 1846G>A (1934G>A) is diagnostic of CYP2D6 *10. Cells transfected with *CYP2D6* 100C>T alone exhibit reduced function, suggesting that this mutation contributes to the reduced function of the CYP2D6 *10 allele. Association studies have examined the role of this variant in contributing to generalized tonic-clonic seizures (GTCS) seen in epilepsy and tardive dyskinesia in Chinese schizophrenic patients [24, 220, 223].

## 12. Major Haplotypes

*CYP2D6*-related major haplotypes include the following.

**CYP2D6^*∗*^1**

*CYP2D6* ^*∗*^ *1* is the reference haplotype for *CYP2D6*. Together with CYP2D6*2, this haplotype forms the group of individuals known as extensive metabolizers or EMs. CYP2D6*1 is usually the majority allele for populations of European and African descent. EMs are considered the norm, and all the other haplotypes are defined as deviations from CYP2D6*1. Population frequencies of this haplotype are African-American: 29–35%; Amerindian: 66%; Central/South Asia: 31%; Chinese: 23%; Colombian: 39%; East Asian: 31%; European: 34%; European Caucasian: 33–36%; Gabonese: 32%; Ghanaian: 44%; Iberian: 56–72%; Japanese: 42-43%; Malay: 36%; Mexican: 57%; Middle Eastern: 35%; Native American: 60%; North African: 12%; Oceanian: 72%; Subsaharan African: 24%; Tanzanian: 28–59%; US Caucasian: 36–40% [220].

**CYP2D6^*∗*^2**

CYP2D6*2 (*CYP2D6*-2850C>T) has slightly reduced function when compared with CYP2D6*1. CYP2D6*1 and CYP2D6*2 represent typical EMs. Population frequencies are African-American: 18–27%; Amerindian: 19%; Central/South Asian: 29%; Chinese: 2%; Colombian: 37%; East Asian: 16%; European: 29%; European Caucasian: 22–33%; Gabonese: 44%; Ghanaian: 11%; Iberian: 2%; Japanese: 9–12%; Mexican: 23%; Middle Eastern: 25%; Native American: 30%; North African: 28%; Oceanian: 0%; Subsaharan African: 33%; Tanzanian: 2%; US Caucasian: 26–37% [220].

**CYP2D6^*∗*^3**

CYP2D6*3 (*CYP2D6*-2549delA) is a completely nonfunctional allele caused by a frameshift mutation that causes a premature truncation of the CYP2D6 protein. CYP2D6*3 is one of several *CYP2D6* haplotypes that can contribute to the phenotypic observation of a poor metabolizer (PM). CYP2D6*3 makes a minor contribution to the poor metabolizer phenotype in Caucasian populations and is virtually nonexistent in non-Caucasian populations. Population frequencies are African-American: 0%; Amerindian: 0%; Central/South Asian: 0%; Chinese: 1%; Colombian: 1.2%; East Asian: 0%; Ethiopian: 0%; European: 0%; European Caucasian: 1–4%; Ghanaian: 0%; Iberian: 2%; Inuit: 0%; Mexican: 1%; Middle Eastern: 0%; Native American: 0%; North African: 0%; Oceanian: 0%; Subsaharan African: 0%; Tanzanian: 0%; US Caucasian: 1-2%; Zimbabwean: 0% [220].

**CYP2D6^*∗*^4**

CYP2D6*4 (*CYP2D6*-100C>T and 1846G>A) is a nonfunctional haplotype that contributes to the majority of PMs in Caucasian populations. CYP2D6*4 carriers are at increased risk when compared to their EM counterparts for toxicities or lack of efficacy due to *CYP2D6* inactivity. Population frequencies are African-American: 6–8%; Amerindian: 4–17%; Central/South Asian: 8%; Chinese: 1%; Colombian: 19.4%; East Asian: 3%; Ethiopian: 1%; European: 17%; European Caucasian: 12–21%; Ghanaian: 7%; Iberian: 13%; Inuit: 8%; Japanese: 1%; Malay: 3%; Mexican: 10%; Middle Eastern: 7%; Native American: 3%; North African: 12%; Oceanian: 0%; Subsaharan African: 3%; Tanzanian: 1%; US Caucasian: 18–23%; Zimbabwean: 2-3% [220].

**CYP2D6^*∗*^5**

CYP2D6*5 results in a nonfunctional haplotype due to a whole gene deletion. This allele contributes to the PM phenotype pool with a frequency of 1–7% in most populations. Population frequencies are African-American: 6-7%; Amerindian: 4%; Central/South Asian: 4%; Chinese: 6%; Colombian: 0.8%; East Asian: 6%; Ethiopian: 3%; European: 3%; European Caucasian: 2–7%; Gabonese: 1%; Ghanaian: 1%; Iberian: 3%; Japanese: 5-6%; Malay: 5%; Mexican: 2%; Middle Eastern: 4%; Native American: 1%; North African: 3%; Oceanian: 1%; Subsaharan African: 6%; Tanzanian: 6%; US Caucasian: 2–5%; Zimbabwean: 4% [220].

**CYP2D6^*∗*^6**

CYP2D6*6 (*CYP2D6-*1707delT) is a nonfunctional haplotype of *CYP2D6*. CYP2D6*6 is caused by a frameshift mutation that results in a truncated and nonfunctional CYP2D6 protein. CYP2D6*6 is found primarily in Caucasian populations. Population frequencies are African-American: 0%; Amerindian: 1%; Central/South Asian: 0%; Colombian: 0%; East Asian: 0%; European: 1%; European Caucasian: 1%; Ghanaian: 0%; Iberian: 3%; Inuit: 8%; Middle Eastern: 1%; Native American: 0%; North African: 0%; Oceanian: 0%; Subsaharan African: 0%; Tanzanian: 0%; US Caucasian: 1% [220].

**CYP2D6^*∗*^9**

CYP2D6*9 (*CYP2D6-*2613–2615delAGA) is a reduced function haplotype caused by the deletion of a single amino acid; its mRNA lacks a single codon resulting in deletion of Lys281. This variant probably represents less than 1.5% of all *CYP2D6 *alleles. The highest frequency is present among Caucasians of Europe and North America. Population frequencies are African-American: 0%; Amerindian: 0%; Central/South Asian: 0%; East Asian: 0%; European: 3%; European Caucasian: 0–2%; Ghanaian: 0%; Malay: 3%; Middle Eastern: 0%; Native American: 0%; North African: 0%; Oceanian: 0%; Subsaharan African: 0%; Tanzanian: 0%; US Caucasian: 2-3%; Zimbabwean: 0% [220].

**CYP2D6^*∗*^10**

CYP2D6*10 (*CYP2D6-*100C>T) is a reduced function haplotype very common in populations of Asian ancestry, especially among Japanese and Malay. Homozygotes of this allele are common and result in the IM phenotype. IMs are also at risk for adverse events and lack of efficacy similar to those seen in PMs, although not as severe, due to the residual activity of CYP2D6*10. Population frequencies are African-American: 3–8%; Amerindian: 2–18%; Central/South Asian: 4%; Chinese: 5–7%; East Asian: 4%; Ethiopian: 9%; European: 3%; European Caucasian: 1-2%; Ghanaian: 3%; Iberian: 2–5%; Inuit: 2%; Japanese: 38–41%; Malay: 50%; Mexican: 7%; Middle Eastern: 1%; Native American: 0%; North African: 0%; Oceanian: 3%; Subsaharan African: 4%; Tanzanian: 4%; US Caucasian: 2–8%; Zimbabwean: 0% [220].

**CYP2D6^*∗*^17**

CYP2D6*17 (*CYP2D6*-2850C>T and 1023C>T) is a reduced function allele of *CYP2D6*. CYP2D6*17 was frequently misdiagnosed as CYP2D6*2 in the early studies of *CYP2D6* genotyping, particularly in populations of African origin. CYP2D6*17 carriers show an IM phenotype. The highest frequency of this haplotype appears in Africans. Population frequencies are African-American: 15–23%; Central/South Asian: 0%; Colombian: 1.7%; East Asian: 0%; Ethiopian: 1%; European: 0%; European Caucasian: 0%; Gabonese: 24%; Ghanaian: 28%; Malay: 1%; Mexican: 1%; Middle Eastern: 2%; Native American: 1%; North African: 8%; Oceanian: 0%; Subsaharan African: 12%; Tanzanian: 17%; US Caucasian: 0%; Zimbabwean: 34% [220].

**CYP2D6^*∗*^29**

CYP2D6*29 (*CYP2D6* 2850C>T and *CYP2D6* 1659G>A and *CYP2D6* 3183G>A) is a reduced functioning haplotype of *CYP2D6*. This haplotype was originally discovered in African populations and contributes towards the IM phenotype of *CYP2D6*. Population frequencies are Central/South Asian: 0%; East Asian: 0%; European: 0%; Middle Eastern: 0%; Native American: 0%; North African: 0%; Oceanian: 0%; Subsaharan African: 7% [220].

**CYP2D6^*∗*^41**

CYP2D6*41 (*CYP2D6*-2850C>T and 2988G>A) is also a reduced functioning haplotype of *CYP2D6* that contributes towards the IM phenotype. Population frequencies are Central/South Asian: 11%; East Asian: 2%; European: 7%; Middle Eastern: 17%; Native American: 0%; North African: 8%; Oceanian: 0%; Subsaharan African: 3% [220].

**CYP2D6-UM**

*CYP2D6* UM is a generic term used to indicate multiple *CYP2D6* copies (2–13) that cause the ultrarapid metabolizer (UM) phenotype. Gene duplications are present in many different *CYP2D6* haplotypes, including CYP2D6*1, CYP2D6*2, CYP2D6*4, CYP2D6*10, and CYP2D6*41. These gene copies can cause a lack of efficacy by quickly metabolizing a parent drug. UMs can suffer from similar problems as PMs, despite having opposite phenotypes. For instance, both can experience a lack of efficacy, but in the case of UMs it would be from quickly metabolizing a parent drug, whereas in the case of PMs it would be the inability to form an active metabolite. Toxicity can occur in both haplotypes. UMs would experience toxicities resulting from a high level of metabolite, whereas the PMs would experience toxicities resulting from a high level of parent drug. Population frequencies of combined CYP2D6*1 and CYP2D6*2 haplotypes are African-American: 1–5%; Amerindian: 3%; Central/South Asian: 1%; Chinese: 1%; Colombian: 1.2%; East Asian: 12%; European: 1%; European Caucasian: 2%; Gabonese: 3%; Iberian: 7%; Middle Eastern: 2%; Native American: 5%; North African: 7%; Oceanian: 5%; Subsaharan African: 28%; Tanzanian: 14%; US Caucasian: 1% [220, 223].

## 13. Phenotypes

The classification of *CYP2D6* phenotypes according to major haplotypes is as follows.

**Extensive Metabolizers (EMs):**

normal enzyme activity: CYP2D6*1, CYP2D6*2, CYP2D6*27, CYP2D6*33, CYP2D6*35, CYP2D6*39, and CYP2D6*48.

**Intermediate Metabolizers (IMs):**

decreased enzyme activity: CYP2D6*9, CYP2D6*10, CYP2D6*17, CYP2D6*29, CYP2D6*41, CYP2D6*49, CYP2D6*50, CYP2D6*51, CYP2D6*55, CYP2D6*59, and CYP2D6*72.

**Poor Metabolizers (PMs):**

no or negligible enzyme activity: CYP2D6*3, CYP2D6*4, CYP2D6*5, CYP2D6*6, CYP2D6*11, CYP2D6*12, CYP2D6*13, CYP2D6*14, CYP2D6*15, CYP2D6*16, CYP2D6*18, CYP2D6*19, CYP2D6*20, CYP2D6*21, CYP2D6*36, CYP2D6*38, CYP2D6*40, CYP2D6*42, CYP2D6*44, CYP2D6*47, CYP2D6*51, CYP2D6*56, CYP2D6*57, and CYP2D6*62.

**Ultrarapid Metabolizers (UMs):**

increased enzyme activity: CYP2D6*1×N, CYP2D6*2×N, CYP2D6*35×*2, *and CYP2D6*53 [24, 220, 222, 223].

## 14. *CYP2D6* GenoPhenotypes in CNS Disorders

Members of most CYP families have been identified in animal and human brains. There is extensive information available on the regional and cellular distribution of most CYP families in rodent brains, but very little is known about the human brain; only *CYP2D6* has been mapped throughout the human brain. An important role ascribed to brain CYPs is the metabolism of endogenous neurally derived or acting compounds, such as neurotransmitters and neurosteroids. Although *CYP2D6* does not have a primary role in the synthesis of dopamine, it may have a modulatory effect on dopamine metabolism in the brain. CYP2D6 was found in close association with the dopamine transporter, CYP2D enzymes have been found in dopaminergic cells in the rat substantia nigra, and CYP2D6 and rat brain-specific CYP2D18 have been implicated in dopamine metabolism [229]. Genetic polymorphisms in *CYP2D6* have been suggested to be associated with smoking behavior, and this modification may occur through the involvement of *CYP2D6* in the dopaminergic pathway. Genetic defects in *CYP2D6* have been associated with Parkinson's disease, which may be linked to the role of *CYP2D6* in dopamine metabolism in the brain [229]. Not only may CYPs contribute to the metabolism of neurotransmitters, but neurotransmitters, their precursors, and their metabolites may have a modulatory effect on the catalytic activity of CYPs in the brain. Tryptamine inhibits CYP2D6-mediated dextromethorphan O-demethylation, serotonin and tryptamine inhibit CYP1A2 phenacetin O-deethylase activity, and 5-hydroxytryptamine and adrenaline inhibit diclofenac 4-hydroxylation by CYP2C9 *in vitro*. The effect of these indoleamines and catecholamines on CYP activity suggests that in the brain local drug metabolism by CYPs may be modulated or regulated by endogenous neurotransmitters, their precursors, or metabolites, and this may play a role in the observed interindividual variability in drug response [229].

*CYP2D6* is involved in the biotransformation of many drugs, which predominantly act in the CNS, including opioids, many psychotropic drugs, and neurotoxins. Until now, however, only controversial information is available regarding the presence of *CYP2D6* in the CNS. The regional and cellular expression of CYP2D6 transcripts and proteins in *postmortem* brain tissues of three individuals was analyzed [230]. Neuronal cells, as well as glial cells, showed labeling for mRNA in brain regions such as the neocortex, caudate nucleus, putamen, globus pallidus, hippocampus, hypothalamus, thalamus, substantia nigra, and cerebellum. In contrast, CYP2D6 protein was primarily localized in large principal neurons such as pyramidal cells of the cortex, pyramidal cells of the hippocampus, and Purkinje cells of the cerebellum. In glial cells, CYP2D6 protein was absent. These results provide clear evidence of CYP2D6 expression in certain regions of the CNS and may indicate the role *CYP2D6* plays in a number of drug interactions that are of potential clinical importance for neurological diseases [230].

**Figure 6:**
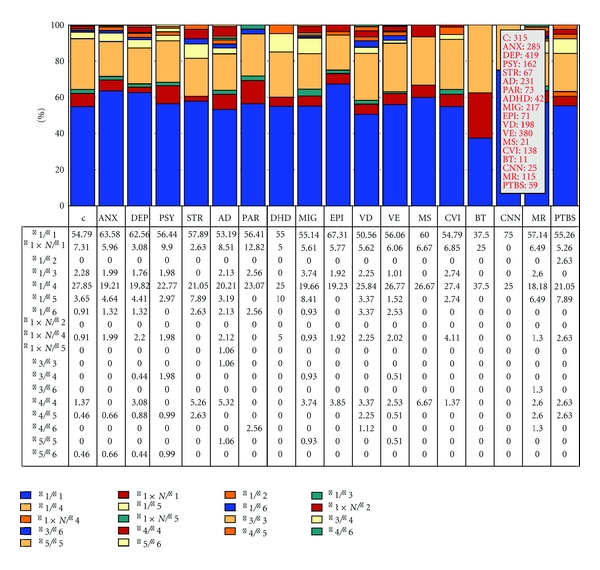
Distribution and frequency of *CYP2D6* variants in patients with CNS disorders. C: controls; ANX: anxiety; DEP: depression; PSY: psychotic disorders; STR: stroke; AD: Alzheimer's disease; PAR: Parkinson's disease; ADHD: attention-deficit hyperactivity disorder; MIG: migraine; EPI: epilepsy; VD: vascular dementia; VE: vascular encephalopathy; MS: multiple sclerosis; CVI: cerebrovascular insufficiency; BT: brain tumors; CNN: cranial nerve neuropathies; MR: mental retardation; PTBS: posttraumatic brain injury syndrome. Source: R. Cacabelos. CIBE Database. EuroEspes Biomedical Research Center, Institute for CNS Disorders, Coruña, Spain.

**Figure 7:**
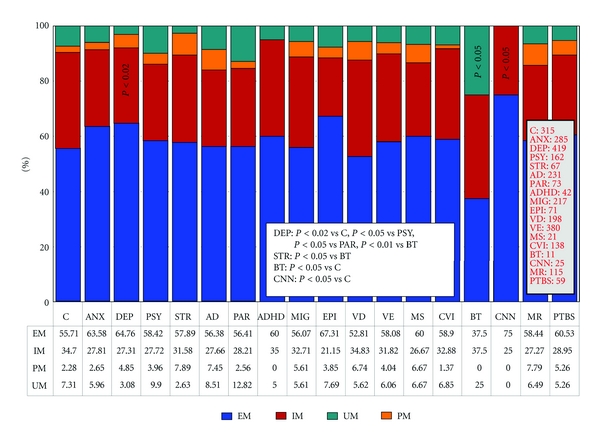
Distribution and frequency of *CYP2D6* extensive metabolizers (EMs), intermediate metabolizers (IMs), poor metabolizers (PMs), and ultrarapid metabolizers (UMs) in patients with different CNS disorders. C: controls; ANX: anxiety; DEP: depression; PSY: psychotic disorders; STR: stroke; AD: Alzheimer's disease; PAR: Parkinson's disease; ADHD: attention-deficit hyperactivity disorder; MIG: migraine; EPI: epilepsy; VD: vascular dementia; VE: vascular encephalopathy; MS: multiple sclerosis; CVI: cerebrovascular insufficiency; BT: brain tumors; CNN: cranial nerve neuropathies; MR: mental retardation; PTBS: posttraumatic brain injury syndrome. Source: R. Cacabelos. CIBE Database. EuroEspes Biomedical Research Center, Institute for CNS Disorders, Coruña, Spain.

The distribution and frequency of *CYP2D6* genotypes (Figure 6) and phenotypes (Figure 7) were investigated in 315 Spanish controls with no family history of neuropsychiatric disorders and in patients with anxiety (*N* = 285), depression (*N* = 419), psychosis (*N* = 162), stroke (*N* = 67), Alzheimer's disease (*N* = 231), Parkinson's disease (*N* = 73), attention-deficit hyperactivity disorder (*N* = 42), migraine (*N* = 217), epilepsy (*N* = 71), vascular dementia (*N* = 198), vascular encephalopathy (with hypertension, diabetes, or dyslipidemia) (*N* = 380), multiple sclerosis (*N* = 21), cerebrovascular insufficiency (*N* = 138), brain tumors (glioma, astrocytoma, glioblastoma, and meningioma) (*N* = 11), cranial nerve neuropathy (facial palsy, and trigeminal neuralgia) (*N* = 25), mental retardation (*N* = 115), and posttraumatic brain injury syndrome (*N* = 59) (Figures 6 and 7). In healthy subjects, EMs accounted for 55.71% of the population, whereas IMs were 34.7%, PMs 2.28%, and UMs 7.31% (Figure 7). Patients with depression showed significant differences in the genotypic and phenotypic profiles as compared to controls (*P* < 0.02) and also with respect to patients with psychosis (*P* < 0.05), Parkinson's disease (*P* < 0.05), or brain tumors (*P* < 0.05). Patients with stroke showed differences as compared to patients with brain tumors (*P* < 0.05), and both patients with brain tumors or with cranial nerve neuropathies differed in their *CYP2D6* phenotype with regard to controls (*P* < 0.05). These genophenotypic profiles might be important in the pathogenesis of some CNS disorders and in the therapeutic response to conventional psychotropic drugs as well (Figures 6 and 7).

## 15. *CYP2D6 *in Alzheimer's Disease

In the Iberian population (Spain + Portugal), where the mixture of ancestral cultures has occurred for centuries, the distribution of the *CYP2D6* genotypes differentiates 4 major categories of *CYP2D6*-related metabolizer types: (i) extensive metabolizers (EMs) (*1/*1,*1/*2,*1/*10), (ii) intermediate metabolizers (IMs) (*1/*3, *1/*4, *1/*5, *1/*6, *1/*7, *10/*10, *4/*10, *6/*10, *7/*10), (iii) poor metabolizers (PMs) (*4/*4, *5/*5), and (iv) ultrarapid metabolizers (UMs) (*1×N/*1, *1×N/*4, Dupl). In our sample we found 51.61% EMs, 32.26% IMs, 9.03% PMs, and 7.10% UMs [4, 6, 7, 9, 28, 112, 113, 115]. In a more recent study with 1,637 subjects and 644 patients with AD we did not find any significant difference between AD cases and the general population (GP) [5]. A variation rate higher than 2% was found only in the EM-*1/*1 genotype, which is more frequent in the GP than in AD. The proportion of EMs was 59.51% in GP and 57.76% in AD; IMs were 29% in GP and 31% in AD; PMs were 4.46% in GP and 5.27% in AD; UMs were 6.23% in GP and 5.9% in AD [5]. No major differences between females and males were found in the GP group; however, in AD, EMs are more frequent in females than in males, and PMs are more frequent in males than in females, indicating that males might be at higher risk for developing ADRs [5].

## 16. Association of *CYP2D6* Variants with Alzheimer's Disease-Related Genes

We have also investigated the association of *CYP2D6* genotypes with AD-related genes, such as *APP*, *MAPT*,* APOE*,* PSEN1*,* PSEN2*,* A2M*,* ACE*,* AGT*,* FOS*, and *PRNP* variants [4–7, 112, 113]. Homozygous *APOE-2/2* (12.56%) and *APOE-4/4* (12.50%) accumulate in UMs, and *APOE-4/4* cases were also more frequent in PMs (6.66%) than in EMs (3.95%) or IMs (0%). *PSEN1-1/1* genotypes were more frequent in EMs (45%), whereas *PSEN-1/2* genotypes were overrepresented in IMs (63.16%) and UMs (60%). The presence of the *PSEN1-2/2* genotype was especially high in PMs (38.46%) and UMs (20%). A mutation in the *PSEN2* gene exon 5 (PS2E5+) was markedly present in UMs (66.67%). About 100% of UMs were *A2M*-V100I-A/A, and the *A2M*-V100I-G/G genotype was absent in PMs and UMs. The *A2M*-I/I genotype was absent in UMs, and 100% of UMs were *A2M*-I/D and *ACE*-D/D. Homozygous mutations in the *FOS* gene (B/B) were also only present in UMs. *AGT*-T235T cases were absent in PMs, and the *AGT*-M174M genotype appeared in 100% of PMs. Likewise, the *PRNP*-M129M variant was present in 100% of PMs and UMs. These association studies clearly show that in PMs and UMs there is an accumulation of AD-related polymorphic variants of risk which might be responsible for the defective therapeutic responses currently seen in these AD clusters [4, 6, 7, 112–115].

## 17. *CYP2D6*-Related Biochemical and Hemodynamic Phenotypes in Alzheimer's Disease

It appears that different *CYP2D6* variants, expressing EMs, IMs, PMs, and UMs, influence to some extent several biochemical parameters, liver function, and vascular hemodynamic parameters, which might affect drug efficacy and safety. Blood glucose levels are found to be elevated in EMs (*1/*1 versus *4/*10) and in some IMs (*4/*10 versus *1×N/*4), whereas other IMs (*1/*5 versus *4/*4) tend to show lower levels of glucose compared with PMs (*4/*4) or UMs (*1×N/*4). The highest levels of total cholesterol are detected in the EMs with the CYP2D6*1/*10 genotype (versus *1/*1, *1/*4, and *1×N/*1). The same pattern has been observed with regard to LDL-cholesterol levels, which are significantly higher in the EM-*1/*10. In general, both total cholesterol levels and LDL-cholesterol levels are higher in EMs (with a significant difference between *1/*1 and *1/*10), intermediate levels are seen in IMs, and much lower levels in PMs and UMs; and the opposite occurs with HDL-cholesterol levels, which on average appear much lower in EMs than in IMs, PMs, and UMs, with the highest levels detected in *1/*3 and *1×N/*4. The levels of triglycerides are highly variable among different *CYP2D6 *polymorphisms, with the highest levels present in IMs (*4/*10 versus *4/*5 and *1×N/*1) [4, 6, 115]. These data clearly indicate that lipid metabolism can be influenced by *CYP2D6 *variants or that specific phenotypes determined by multiple lipid-related genomic clusters are necessary to confer the character of EMs and IMs. Another possibility might be that some lipid metabolism genotypes interact with CYP2D6-related enzyme products, leading to the definition of the pheno-genotype of PMs and UMs. No significant changes in blood pressure values have been found among *CYP2D6 *genotypes; however, important differences became apparent in brain cerebrovascular hemodynamics. The best cerebrovascular hemodynamic pattern is observed in EMs and PMs, with higher brain blood flow velocities and lower resistance and pulsatility indices, but differential phenotypic profiles are detectable among *CYP2D6* genotypes. Systolic blood flow velocities (Sv) in the left middle cerebral arteries (LMCA) of AD patients are significantly lower in *1/*10 EMs, with high total cholesterol and LDL-cholesterol levels, than in IMs (*4/*10); diastolic velocities (Dv) also tend to be much lower in *1/*10 and especially in PMs (*4/*4) and UMs (*1×N/*4), whereas the best Dv is measured in *1/*5 IMs. More striking are the results of both the pulsatility index (PI = (Sv − Dv)/Mv) and resistance index (RI = (Sv − Dv)/Sv), which are worse in IMs and PMs than in EMs and UMs. These data taken together seem to indicate that *CYP2D6*-related AD PMs exhibit a poorer cerebrovascular function which might affect drug penetration into the brain, with the consequent therapeutic implications [4, 6, 7, 112–115].

## 18. Influence of *CYP2D6* Genotypes on Liver Transaminase Activity

UMs and PMs tend to show the highest GOT activity and IMs the lowest. Significant differences appear among different IM-related genotypes. The *10/*10 genotype exhibited the lowest GOT activity with marked differences as compared to UMs. GPT activity was significantly higher in PMs (*4/*4) than in EMs (*1/*10) or IMs (*1/*4 and*1/*5). The lowest GPT activity was found in EMs and IMs. Striking differences have been found in GGT activity between PMs (*4/*4), which showed the highest levels, and EMs (*1/*1 and *1/*10), IMs (*1/*5), or UMs (*1×N/*1) [6]. Interesting enough, the *10/*10 genotype, with the lowest values of GOT and GPT, exhibited the second highest levels of GGT after *4/*4, probably indicating that *CYP2D6*-related enzymes differentially regulate drug metabolism and transaminase activity in the liver. These results are also clear in demonstrating the direct effect of *CYP2D6* variants on transaminase activity [4, 6, 7].

## 19. *CYP2D6*-Related Therapeutic Response to a Multifactorial Treatment in Dementia

With a multifactorial therapeutic intervention in patients with dementia who received (a) an endogenous nucleotide and choline donor, CDP-choline (500 mg/day), (b) a nootropic substance, piracetam (1600 mg/day), (c) a vasoactive compound, 1,6 dimethyl 8*β*-(5-bromonicotinoyl-oxymethyl)-10*α*-methoxyergoline (nicergoline) (5 mg/day), and (d) a cholinesterase inhibitor, donepezil (5 mg/day), for one year, EMs improved their cognitive function (MMSE score) from 21.58 ± 9.02 at baseline to 23.78 ± 5.81 after 1-year treatment. IMs also improved from 21.40 ± 6.28 to 22.50 ± 5.07 (*r* = +0.96), whereas PMs and UMs deteriorated from 20.74 ± 6.72 to 18.07 ± 5.52 (*r* = −0.97) and from 22.65 ± 6.76 to 21.28 ± 7.75 (*r* = −0.92), respectively. According to these results, PMs and UMs were the worst responders, showing a progressive cognitive decline with no therapeutic effect, and EMs and IMs were the best responders, with a clear improvement in cognition after one year of treatment. Among EMs, AD patients harboring the *1/*10 genotype responded better than patients with the *1/*1 genotype. The best responders among IMs were the *1/*3, *1/*6, and *1/*5 genotypes, whereas the *1/*4, *10/*10, and *4/*10 genotypes were poor responders. Among PMs and UMs, the poorest responders were carriers of the *4/*4 and *1×N/*1 genotypes, respectively [4–7, 9, 28, 112, 113]. In a recent study, Pilotto et al. [231] have confirmed the influence of *CYP2D6 *variants (rs1080985) on the efficacy of donepezil in AD.

From all these data in patients with dementia we can conclude the following. (i) The most frequent *CYP2D6 *variants in the Southern European population (Iberian peninsula) are the *1/*1 (57.84%), *1/*4 (22.78%), *1×N/*1 (6.10%), *4/*4 (2.56%), and *1/*3 (2.01%) genotypes, accounting for more than 80% of the population; (ii) the frequency of EMs, IMs, PMs, and UMs is about 59.51%, 29.78%, 4.46%, and 6.23%, respectively, in the general population, and 57.76, 31.05%, 5.27%, and 5.90%, respectively, in AD cases; (iii) EMs are more prevalent in GP (59.51%) than in AD (57.76%); IMs are more frequent in AD (31.05%) than in GP (29.78%); the frequency of PMs is slightly higher in AD (5.27%) than in GP (4.46%); UMs are more frequent in GP (6.23%) than in AD (5.90%); (iv) there are differences between females and males in the distribution and frequency of *CYP2D6* genotypes, which might be of relevance in therapeutic terms and risk of ADRs; (v) there is an accumulation of AD-related genes of risk in PMs and UMs; (vi) PMs and UMs tend to show higher transaminase activities than EMs and IMs; (vii) EMs and IMs are the best responders, and PMs and UMs are the worst responders to a combination therapy with cholinesterase inhibitors, neuroprotectants, and vasoactive substances; (viii) the pharmacogenetic response in AD appears to be dependent upon the networking activity of genes involved in drug metabolism and genes involved in AD pathogenesis [4–7, 9, 28, 105, 112, 113].

## 20. *CYP* Clustering

Since over half of the available drugs are metabolized via different CYP enzymes and other metabolic pathways, it is convenient to understand the networking activity of *CYP* genes and the genomic profiles of these genes in particular groups of risk. In the case of dementia, 73.71% of AD patients are *CYP2C19*-EMs, 25.12% IMs, and 1.16% PMs. The distribution and frequency of *CYP2C9* genotypes is as follows: *1/*1-EM 60.87%, *1/*2-IM 23.98%, *1/*3-IM 10.17%, *2/*2-PM 2.54%, *2/*3-PM 2.16%, and *3/*3-PM 0.25%, globally representing 60.87% *CYP2C9*-EMs, 34.16% IMs, and 4.97% PMs [5]. This is especially important because the *CYP2C9*-Ile359Leu (CYP2C9*3 allele) and *CYP2C9*-Arg144Cys (CYP2C9*2 allele) variants are associated with warfarin sensitivity. Clustering together *CYP2C9* and *VKORC1* variants, we can estimate that approximately 30% of the elderly population is sensitive to warfarin anticoagulants.

Concerning *CYP3A4/5* polymorphisms, 82.75% of AD cases are EMs (CYP3A5*3/*3), 15.88% are IMs (CYP3A5*1/*3), and 1.37% are UMs (CYP3A5*1/*1) [5].

The construction of a genetic map integrating the most prevalent *CYP2D6 *+ *CYP2C19 *+ *CYP2C9* polymorphic variants in a trigenic cluster yields 82 different haplotype-like profiles. The most frequent trigenic genotypes in the AD population are *1*1-*1*1-*1*1 (25.70%), *1*1-*1*2-*1*2 (10.66%), *1*1-*1*1-*1*1 (10.45%), *1*4-*1*1-*1*1 (8.09%), *1*4-*1*2-*1*1 (4.91%), *1*4-*1*1-*1*2 (4.65%), and *1*1-*1*3-*1*3 (4.33%). These 82 trigenic genotypes represent 36 different pharmacogenetic phenotypes. According to these trigenic clusters, only 26.51% of the patients show a pure 3EM phenotype, 15.29% are 2EM1IM, 2.04% are pure 3IM, 0% are pure 3PM, and 0% are 1UM2PM (the worst possible phenotype) [5].

Taking into consideration the data available, it might be inferred that at least 10–15% of the AD population may exhibit an abnormal metabolism of cholinesterase inhibitors and/or other drugs, which undergo oxidation via *CYP2D6*-related enzymes. Approximately 50% of this population cluster would show an ultrarapid metabolism, requiring higher doses of cholinesterase inhibitors in order to reach a therapeutic threshold, whereas the other 50% of the cluster would exhibit a poor metabolism, displaying potential adverse events at low doses. If we take into account that approximately 60–70% of therapeutic outcomes depend upon pharmacogenomic criteria (e.g., pathogenic mechanisms associated with AD-related genes), it can be postulated that pharmacogenetic and pharmacogenomic factors are responsible for 75–85% of the therapeutic response (efficacy) in AD patients treated with conventional drugs [7, 9, 105, 112, 113].

## 21. Conclusions

Major conclusions to be drawn from studies on AD genomics and pharmacogenomics would be the followung: (i) AD is a complex disorder in which many different gene clusters may be involved; (ii) most genes screened to date belong to different proteomic and metabolomic pathways potentially affecting AD pathogenesis; (iii) the *APOE* gene seems to be a major risk factor for both degenerative and vascular dementia; (iv) the therapeutic response to conventional drugs in patients with AD is genotype-specific, with *CYP2D6*-PMs, *CYP2D6*-UMs, and *APOE-4/4 *carriers acting as the worst responders; (v) *APOE* and *CYP2D6* may cooperate, as pleiotropic genes, in the metabolism of drugs and hepatic function; (vi) the introduction of pharmacogenetic procedures into AD pharmacological treatment may help to optimize therapeutics.

## Figures and Tables

**Table 1:** Selected human genes investigated as potential candidate genes associated with dementia and age-related neurodegenerative disorders [42].

Locus	Symbol	Aliases	Title
1p21.3-p13.1	*SORT1*	*Gp95, NT3*	Sortilin
1p31.3	*TM2D1*	*BBP*	TM2 domain containing 1
1p32	*ERI3*	*PINT1; PRNPIP; MGC2683; FLJ22943*	ERI1 exoribonuclease family member 3
1p32.3	*ZFYVE9*	*MADHIP, NSP, SARA, SMADIP*	Zinc finger, FYVE domain containing 9
1p33-p31.1	*DHCR24*	*KIAA0018, Nbla03646, SELADIN1, seladin-1*	24-dehydrocholesterol reductase
1p34	*LRP8*	*APOER2, HSZ75190, MCI1*	Low-density lipoprotein receptor-related protein 8, apolipoprotein E receptor
1p36.1	*ECE1*	*RP3-329E20.1, ECE*	Endothelin-converting enzyme 1
1p36.13-q31.3	*APH1A*	*RP4-790G17.3, 6530402N02Rik, APH-1, APH-1A, CGI-78*	Anterior pharynx defective 1 homolog A (*C. elegans*)
1p36.22	*TARDBP*	*RP4-635E18.2, ALS10, TDP-43*	TAR DNA-binding protein
1p36.3	*MTHFR*		5,10-methylenetetrahydrofolate reductase (NADPH)
1q21	*S100A1*	*S100, S100-alpha, S100A*	S100 calcium-binding protein A1
1q21.2-q21.3	*LMNA*	*RP11-54H19.1, CDCD1, CDDC, CMD1A, CMT2B1, EMD2, FPL, FPLD, HGPS, IDC, LDP1, LFP, LGMD1B, LMN1, LMNC, PRO1*	Lamin A/C
1q21.3	*CHRNB2*	*EFNL3, nAChRB2*	Cholinergic receptor, nicotinic, beta 2 (neuronal)
1q21-q23	*APCS*	*MGC88159, PTX2, SAP*	Amyloid P component, serum
1q22-q23	*NCSTN*	*RP11-517F10.1, APH2, KIAA0253*	Nicastrin
1q25	*SOAT1*	*RP11-215I23.1, ACACT, ACAT, ACAT1, RP11-215I23.2, SOAT, STAT*	Sterol O-acyltransferase 1
1q25.2-q25.3	*PTGS2*	*COX-2, COX2, GRIPGHS, PGG/HS, PGHS-2, PHS-2, hCox-2*	Prostaglandin-endoperoxide synthase 2 (prostaglandin G/H synthase and cyclooxygenase)
1q31-q32	*IL10 *	*CSIF, IL-10, IL10A, MGC126450, MGC126451, TGIF*	Interleukin-10
1q31-q42	*AD4*	*AD3L, AD4, PS2, STM2*	Presenilin 2 (Alzheimer's disease 4)
1q32	*CR1*	*C3BR, C4BR, CD35, KN*	Complement component (3b/4b) receptor 1 (Knops blood group)
1q42-q43	*AGT*	*ANHU, FLJ92595, FLJ97926, SERPINA8*	Angiotensinogen (serpin peptidase inhibitor, clade A, member 8)
2p16.3	*RTN4 *	*ASY, NI220/250, NOGO, NOGO-A, NOGOC, NSP, NSP-CL, Nbla00271, Nbla10545, Nogo-B, Nogo-C, RTN-X, RTN4-A, RTN4-B1, RTN4-B2, RTN4-C*	Reticulon 4
2p25	*ADAM17*	*ADAM18, CD156B, CSVP, MGC71942, TACE*	ADAM metallopeptidase domain 17
2q14	*BIN1*	*AMPH2, AMPHL, DKFZp547F068, MGC10367, SH3P9*	Bridging integrator 1
2q14	*IL1A*	*IL-1A, IL1, IL1-ALPHA, IL1F1*	Interleukin-1-Alpha
2q21.1	*KCNIP3*	*CSEN, DREAM, KCHIP3, MGC18289*	Kv channel interacting protein 3, calsenilin
2q21.2	*LRP1B*	*LRP-DIT, LRPDIT*	Low-density lipoprotein-related protein 1B (deleted in tumors)
2q34	*CREB1*	*CREB, MGC9284*	cAMP responsive element binding protein 1
3q25.1-q25.2		*CALLA, CD10, MME DKFZp686O16152, MGC126681, MGC126707, NEP*	Membrane metalloendopeptidase
3q26.1-q26.2	*BCHE*	*CHE1, E1*	Butyrylcholinesterase
3q26.2-qter		*APOD*	Apolipoprotein D
3q28	*SST*	*SMST*	Somatostatin
4p14-p13	*APBB2*	*DKFZp434E033, FE65L, FE65L1, MGC35575*	Amyloid beta (A4) precursor protein-Binding, family B, member 2
5q15	*CAST*	*BS-17, MGC9402*	Calpastatin
5q31	*APBB3*	*FE65L2, MGC150555, MGC87674, SRA*	Amyloid beta (A4) precursor protein-binding, family B, member 3
5q35.3	*DBN1*	*D0S117E, DKFZp434D064*	Drebrin 1
6p12	*VEGFA*	*RP1-261G23.1, MGC70609, MVCD1, VEGF, VPF*	Vascular endothelial growth factor A
6p21.3	*AGER*	*DAMA-358M23.4, MGC22357, RAGE*	Advanced glycosylation end product-specific receptor
6p21.3	*HFE*	*HFE1, HH, HLA-H, MGC103790, MVCD7, dJ221C16.10.1*	Hemochromatosis
6p21.3	*HLA-A*	*DAQB-90C11.16, Aw-68, Aw-69, FLJ26655, HLAA*	Major histocompatibility complex, class I, A
6p21.3	*TNF*	*DADB-70P7.1, DIF, TNF-alpha, TNFA, TNFSF2*	Tumor necrosis factor (TNF superfamily, member 2)
6p22.1	*PGDB1*	*HUCEP-4, SCAND4, dJ874C20.4*	PiggyBac transposable element derived 1
6p23	*ATXN1*	*ATX1, D6S504E, SCA1*	Ataxin 1
7p21	*IL6*	*BSF2, HGF, HSF, IFNB2, IL-6*	Interleukin-6 (interferon, beta 2)
7q21.3	*PON1 *	*ESA, MVCD5, PON*	Paraoxonase 1
7q22	*RELN*	*PRO1598, RL*	Reelin
7q36	*AD10*		Alzheimer's disease 10
7q36	*NOS3*	*ECNOS, eNOS*	Nitric oxide synthase 3 (endothelial cell)
7q36	*PAXIP1*	*CAGF28, CAGF29, FLJ41049, PACIP1, PAXIP1L, PTIP, TNRC2*	PAX-interacting (with transcription-activation domain) protein 1
8p21-p12	*CLU*	*AAG4, APOJ, CLI, KUB1, MGC24903, SGP-2, SGP2, SP-40, TRPM-2, TRPM2*	Clusterin
8p22	*CTSB*	*APPS, CPSB*	Cathepsin B
9p24.1	*IL33*	*C9orf26, DKFZp586H0523, DVS27, NF-HEV, NFEHEV, RP11-575C20.2*	Interleukin 33
9q13-q21.1	*APBA1*	*D9S411E, MINT1, X11, X11A, X11ALPHA*	Amyloid beta (A4) precursor protein-binding, family A, member 1
9q31.1	*GRIN3A*	*FLJ45414, NMDAR-L, NR3A*	Glutamate receptor, ionotropic, N-methyl-D-aspartate 3A
9q33-q34.1	*HSPA5*	*BIP, FLJ26106, GRP78, MIF2*	Heat shock 70 kDa protein 5 (glucose-regulated protein, 78 kDa)
9q34.1	*DAPK1*	*DAPK, DKFZp781I035*	Death-associated protein kinase 1
10p13	*AD7*		Alzheimer's disease 7
10p15.2	*PITRM1*	*RP11-298E9.1, KIAA1104, MGC138192, MGC141929, MP1, PreP, hMP1*	Pitrilysin metallopeptidase 1
10q	*AD6*		Alzheimer's disease 6
10q11.2	*ALOX5*	*RP11-67C2.3, 5-LO, 5-LOX, 5LPG, LOG5, MGC163204*	Arachidonate 5-lipoxygenase
10q21	*TFAM*	*MtTF1, TCF6, TCF6L1, TCF6L2, TCF6L3, mtTFA*	Transcription factor A, mitochondrial
10q23	*CH25H*	*C25H*	Cholesterol 25-hydroxylase
10q23-q25	*IDE*	*RP11-366I13.1, FLJ35968, INSULYSIN*	Insulin-degrading enzyme
10q23-q25	*SORCS1*	*RP11-446H13.1, FLJ41758, FLJ43475, FLJ44957*	Sortilin-related VPS10 domain containing receptor 1
10q23.32	*HECTD2 *	*FLJ16050*	HECT domain containing 2
10q24	*COX15*		COX15 homolog, cytochrome c oxidase assembly protein (yeast)
10q24	*PLAU*	*ATF, UPA, URK, u-PA*	Plasminogen activator, urokinase
10q24.33	*CALHM1*	*FAM26C, MGC39514, MGC39617*	Calcium homeostasis modulator 1
10q24.33	*SH3PXD2A*	*FISH, SH3MD1*	SH3 and PX domains 2A
10q26.3	*ADAM12 *	*RP11-295J3.5, MCMP, MCMP Mltna, MLTN, MLTNA*	ADAM metallopeptidase domain 12
11p13	*BDNF*	*MGC34632*	Brain-derived neurotrophic factor
11p15	*APBB1*	*FE65, MGC: 9072, RIR*	Amyloid beta (A4) precursor protein-binding, family B, member 1 (Fe65)
11p15.1	*SAA1*	*MGC111216, PIG4, SAA, TP53I4*	Serum amyloid A1
11p15.5	*CTSD*	*CLN10, CPSD, MGC2311*	Cathepsin D
11q14	*PICALM*	*CALM, CLTH, LAP*	Phosphatidylinositol binding clathrin assembly protein
11q14.1	*GAB2 *	*KIAA0571*	GRB2-associated binding protein 2
11q23.2-q23.3	*BACE1*	*ASP2, BACE, FLJ90568, HSPC104, KIAA1149*	Beta-site APP-cleaving enzyme 1
11q23.2-q24.2	*SORL1*	*C11orf32, FLJ21930, FLJ39258, LR11, LRP9, SORLA, SorLA-1, gp250*	Sortilin-related receptor, L(DLR class) A repeats-containing
11q24	*APLP2*	*APPH, APPL2, CDEBP*	Amyloid beta (A4) precursor-like protein 2
12p11.23-q13.12	*AD5*		Alzheimer's disease 5
12p12.3-p12.1	*IAPP*	*AMYLIN, DAP, IAP*	Islet amyloid polypeptide
12p13.3-p12.3	*A2M*	*CPAMD5, DKFZp779B086, FWP007, S863-7*	Alpha-2-macroglobulin
12q13-q14	*LRP1*	*A2MR, APOER, APR, CD91, FLJ16451, IGFBP3R, LRP, MGC88725, TGFBR5*	Low-density lipoprotein-related protein 1 (alpha-2-macroglobulin receptor)
13q34	*DAOA *	*G72, LG72, SG72*	D-amino acid oxidase activator
14q24.3	*FOS*	*AP-1, C-FOS*	FBJ murine osteosarcoma viral oncogene homolog
14q24.3	*PSEN1*	*AD3, FAD, PS1, S182*	Presenilin-1
14q32	*RAGE*	*MOK, RAGE1*	Renal tumor antigen
14q32.1	*CYP46A1*	*CP46, CYP46*	Cytochrome P450, family 46, subfamily A, polypeptide 1
14q32.1	*SERPINA3*	*AACT, ACT, GIG24, GIG25, MGC88254*	Serpin peptidase inhibitor, clade A (alpha-1 antiproteinase, antitrypsin), member 3
15q21.1	*CYP19A1*	*ARO, ARO1, CPV1, CYAR, CYP19, MGC104309, P-450AROM*	Cytochrome P450, family 19, Subfamily A, polypeptide 1
15q22.2	*APH1B*	*APH-1B, DKFZp564D0372, FLJ33115, PRO1328, PSFL, TAAV688*	Anterior pharynx defective 1 homolog B (*C. elegans*)
15q11-q12	*APBA2*	*D15S1518E, HsT16821, LIN-10, MGC99508, MGC: 14091, MINT2, X11L*	Amyloid beta (A4) precursor protein-binding, family A, member 2
16p13.3	*UBE2I *	*C358B7.1, P18, UBC9*	Ubiquitin-conjugating enzyme E2I (UBC9 homolog, yeast)
16q21	*CETP*	*HDLCQ10*	Cholesteryl ester transfer protein, plasma
16q22	*NAE1*	*A-116A10.1, APPBP1, HPP1, ula-1*	NEDD8 activating enzyme E1 subunit 1
17p12-p11.2	*COX10*		COX10 homolog, cytochrome C oxidase assembly protein, heme A: farnesyltransferase (yeast)
17p13	*MYH13*	*MyHC-eo*	Myosin, heavy chain 13, skeletal muscle
17p13.1	*TNK1*	*MGC46193*	Tyrosine kinase, nonreceptor, 1
17q11.2	*BLMH*	*BH, BMH*	Bleomycin hydrolase
17q11.2	*MIR144*	*MIRN144*	MicroRNA 144
17q21.1	*MAPT*	*DDPAC, FLJ31424, FTDP-17, MAPTL, MGC138549, MSTD, MTBT1, MTBT2, PPND, TAU*	Microtubule-associated protein tau
17q21.1	*STH*	*MAPTIT, MGC163191, MGC163193*	Saitohin
17q21.32	*GRN*	*GEP, GP88, PCDGF, PEPI, PGRN*	Granulin
17q21-q22	*GPSC*		Gliosis, familial progressive subcortical
17q21-q23	*APPBP2*	*HS.84084, KIAA0228, PAT1*	Amyloid beta precursor protein (cytoplasmic tail) binding protein 2
17q23.1	*MPO*		Myeloperoxidase
17q23.3	*ACE*	*ACE1, CD143, DCP, DCP1, MGC26566, MVCD3*	Angiotensin-I-converting enzyme (peptidyl-dipeptidase A) 1
17q24.3	*BPTF*	*FAC1, FALZ, NURF301*	Bromodomain PHD finger transcription factor
18q12.1	*TTR*	*HsT2651, PALB, TBPA*	Transthyretin
19p13	*PIN1 *	*DOD, UBL5*	Peptidylprolyl cis/trans isomerase, NIMA-interacting 1
19p13.2	*AD9*		Alzheimer's disease 9
19p13.2-p13.1	*NOTCH3*	*CADASIL, CASIL*	Notch homolog 3 (Drosophila)
19p13.3	*APBA3*	*MGC: 15815, X11L2, mint3*	Amyloid beta (A4) precursor protein-binding, family A, member 3
19p13.3	*GRIN3B *	*NR3B*	Glutamate receptor, ionotropic, N-methyl-D-aspartate 3B
19p13.3-p13.2	*ICAM*	*BB2, CD54, P*3.58	Intercellular adhesion molecule 1
19q13	*TOMM40*	*C19orf1, D19S1177E, PER-EC1, PEREC1, TOM40*	Translocase of outer mitochondrial membrane 40 homolog (yeast)
19q13.1	*APLP1*	*APLP*	Amyloid beta (A4) precursor-like protein 1
19q13.12	*PEN2*	*MDS033, MSTP064, PEN-2, PEN2*	Presenilin enhancer 2 homolog (C. elegans)
19q13.2	*APOE*	*AD2, LDLCQ5, LPG, MGC1571*	Apolipoprotein E
19q13.2	*APOC1*		Apolipoprotein C-I
19q13.32	*BLOC1S3*	*BLOS3, FLJ26641, FLJ26676, HPS8, RP*	Biogenesis of lysosomal organelles complex-1, subunit 3
19q13.32	*EXOC3L2*	*FLJ36147, MGC16332, XTP7*	Exocyst complex component 3-like 2
19q13.3	*MARK4*	*FLJ90097, KIAA1860, MARKL1, Nbla00650*	MAP/microtubule affinity-regulating kinase 4
19q13.43	*GALP*		Galanin-like peptide
20p	*AD8*		Alzheimer's disease 8
20p11.21	*CST3*	*ARMD11, MGC117328*	Cystatin C
20p13	*PRNP*	*ASCR, CD230, CJD, GSS, MGC26679, PRIP, PrP, PrP27-30, PrP33-35C, PrPc, prion*	Prion protein
20q13.31	*PCK1*	*MGC22652, PEPCK-C, PEPCK1, PEPCKC*	Phosphoenolpyruvate carboxykinase 1 (soluble)
21q21.3	*APP*	*AAA, ABETA, ABPP, AD1, APPI, CTFgamma, CVAP, PN2*	Amyloid beta (A4) precursor protein
21q22.3	*BACE2*	*AEPLC, ALP56, ASP1, ASP21, BAE2, CDA13, CEAP1, DRAP*	Beta-site APP-cleaving enzyme 2
22q11.21	*RTN4R*	*NGR, NOGOR*	Reticulon 4 receptor
22q11.21	*COMT*		Catechol-O-methyltransferase
